# Therapeutic Potential of STE20-Type Kinase STK25 Inhibition for the Prevention and Treatment of Metabolically Induced Hepatocellular Carcinoma

**DOI:** 10.1016/j.jcmgh.2025.101485

**Published:** 2025-02-28

**Authors:** Ying Xia, Mara Caputo, Emma Andersson, Bernice Asiedu, Jingjing Zhang, Wei Hou, Manoj Amrutkar, Emmelie Cansby, Nadia Gul, Anne Gemmink, Caitlyn Myers, Mariam Aghajan, Sheri Booten, Andrew J. Hoy, Anetta Härtlova, Per Lindahl, Anders Ståhlberg, Gert Schaart, Matthijs K.C. Hesselink, Andreas Peter, Sue Murray, Margit Mahlapuu

**Affiliations:** 1 Department of Chemistry and Molecular Biology, University of Gothenburg and Sahlgrenska University Hospital, Gothenburg, Sweden; 2 Department of Pathology, Oslo University Hospital Rikshospitalet, Oslo, Norway; 3 Wallenberg Centre for Molecular and Translational Medicine, University of Gothenburg, Gothenburg, Sweden; 4 Sahlgrenska Center for Cancer Research, Department of Surgery, Institute of Clinical Sciences, University of Gothenburg, Gothenburg, Sweden; 5 Department of Nutrition and Movement Sciences, NUTRIM School of Nutrition and Translational Research in Metabolism, Maastricht University Medical Centre, Maastricht, the Netherlands; 6 Department of Microbiology and Immunology, Institute of Biomedicine, University of Gothenburg, Gothenburg, Sweden; 7 Ionis Pharmaceuticals, Carlsbad, California; 8 School of Medical Sciences, Charles Perkins Centre, University of Sydney, Sydney, Australia; 9 Wallenberg Laboratory, Department of Molecular and Clinical Medicine, Institute of Medicine, University of Gothenburg, Gothenburg, Sweden; 10 Department of Biochemistry, Institute of Biomedicine, University of Gothenburg, Gothenburg, Sweden; 11 Sahlgrenska Center for Cancer Research, Department of Laboratory Medicine, Institute of Biomedicine, University of Gothenburg, Gothenburg, Sweden; 12 Department of Clinical Genetics and Genomics, Sahlgrenska University Hospital, Gothenburg, Sweden; 13 Department for Diagnostic Laboratory Medicine, Institute for Clinical Chemistry and Pathobiochemistry, University Hospital Tübingen, Tübingen, Germany; 14 German Center for Diabetes Research (DZD), Neuherberg, Germany; 15 Institute for Diabetes Research and Metabolic Diseases of the Helmholtz Center Munich at the University of Tübingen, Tübingen, Germany; 16 Current affiliation: Shanghai Institute of Transplantation, Renji Hospital, Shanghai Jiao Tong University School of Medicine, Shanghai, China

**Keywords:** Antisense Oligonucleotide Therapy, Hepatocellular Carcinoma, Metabolic Dysfunction-associated Steatohepatitis, STK25

## Abstract

**Background & Aims:**

Hepatocellular carcinoma (HCC) is a rapidly growing malignancy with high mortality. Recently, metabolic dysfunction-associated steatohepatitis (MASH) has emerged as a major HCC catalyst; however, signals driving transition of MASH to HCC remain elusive and treatment options are limited. Herein, we investigated the role of STE20-type kinase STK25, a critical regulator of hepatocellular lipotoxic milieu and MASH susceptibility, in the initiation and progression of MASH-related HCC.

**Methods:**

The clinical relevance of STK25 in HCC was assessed in publicly available datasets and by RT-qPCR and proximity ligation assay in a validation cohort. The functional significance of STK25 silencing in human hepatoma cells was evaluated in vitro and in a subcutaneous xenograft mouse model. The therapeutic potential of STK25 antagonism was examined in a mouse model of MASH-driven HCC, induced by a single diethylnitrosamine injection combined with a high-fat diet.

**Results:**

Analysis of public databases and in-house cohorts revealed that STK25 expression in human liver biopsies positively correlated with HCC incidence and severity. The in vitro silencing of STK25 in human hepatoma cells suppressed proliferation, migration, and invasion with efficacy comparable to that achieved by anti-HCC drugs sorafenib or regorafenib. STK25 knockout in human hepatoma cells also blocked tumor formation and growth in a subcutaneous xenograft mouse model. Furthermore, pharmacologic inhibition of STK25 with antisense oligonucleotides—administered systemically or hepatocyte-specifically—efficiently mitigated the development and exacerbation of hepatocarcinogenesis in a mouse model of MASH-driven HCC.

**Conclusion:**

This study underscores STK25 antagonism as a promising therapeutic strategy for the prevention and treatment of HCC in the context of MASH.

## Graphical abstract

**Figure:**
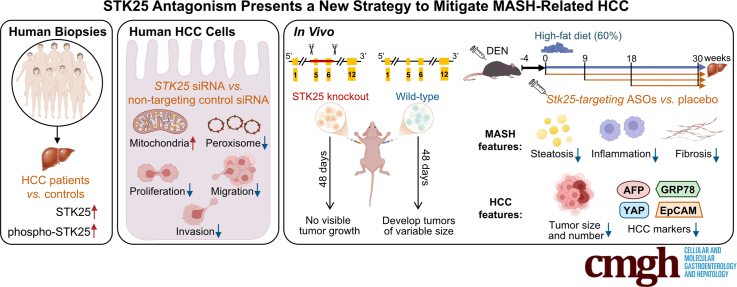

**Summary**

Pharmacologic STK25 antagonism presents a potential prophylactic and therapeutic strategy to mitigate metabolically induced hepatocellular carcinoma: nonclinical proof-of-principle study.

Hepatocellular carcinoma (HCC) is the third leading cause of cancer death globally and the malignancy that displays the steepest increase in both incidence and mortality in the Western world.^1^, ^2^, ^3^ Despite the application of various locoregional and systemic therapies, most individuals with HCC develop disease progression with a low 5-year survival rate of <20% and a high recurrence rate of about 80%.^4^ Recently, the trend in HCC occurrence has shifted epidemiologically from subjects with virus-related liver disease to those with metabolic dysfunction-associated steatohepatitis (MASH; formerly known as non-alcoholic steatohepatitis [NASH]).^4^, ^5^, ^6^ The initial association between MASH and HCC was documented in 2001, and to date, MASH has been reported in several studies as the single most common and rapidly rising underlying etiology of HCC.^1^^,^^2^^,^^7^^,^^8^ Importantly, MASH-related HCC is less likely to be detected and treated less effectively compared with viral infection-induced HCC.^2^^,^^9^

MASH is the advanced subtype of metabolic dysfunction-associated steatotic liver disease (MASLD; previously referred to as non-alcoholic fatty liver disease [NAFLD]), which is characterized by hepatic inflammation, fibrosis, and cell damage in the form of ballooning degeneration and apoptosis, in addition to liver fat infiltration.^5^^,^^10^ Following the obesity epidemics, MASLD has become the most prevalent chronic liver disease, afflicting about 30% of the global population.^10^^,^^11^ Notably, approximately 20% of all individuals with MASLD are expected to demonstrate MASH histology, and progression from MASH to HCC occurs at an estimated rate of 2% per year.^9^ Despite the high clinical relevance, the molecular mechanisms governing the disease exacerbation from MASLD to MASH and further transition to HCC remain elusive.

Our recent studies have identified the liver lipid droplet-decorating protein STK25 (serine/threonine protein kinase 25; also known as YSK1 or SOK1) as a critical driver of the hepatocellular lipotoxic milieu and MASH susceptibility. We have reported that: (1) STK25 mRNA and protein levels positively correlate with the severity of MASH in human liver biopsies^12^, ^13^, ^14^; (2) STK25 knockdown in cultured human hepatocytes reduces ectopic fat accumulation by causing a shift from lipid anabolism towards catabolism^12^^,^^13^^,^^15^; and (3) genetic and pharmacologic inactivation of STK25 in mice ameliorates the full spectrum of diet-induced MASH, including protection against liver steatosis, inflammation, and fibrosis.^12^^,^^14^^,^^16^^,^^17^ In addition, we found that the global knockout of the *STK25* gene efficiently suppresses liver tumor development in 2 mouse models of hepatocarcinogenesis that closely recapitulate the nature of human MASH-driven HCC.^18^ In light of these observations, we now hypothesize that STK25 antagonists could represent an effective therapeutic strategy for the prevention and/or treatment of HCC in the context of MASH.

Here, we performed an in vivo proof-of-principle study to assess the benefits of *Stk25*-targeting antisense oligonucleotides (ASOs), which were either designed to be hepatocyte-specific or broadly distributed to peripheral organs, in blocking the initiation and progression of MASH-related HCC in mice, when administered at different phases of the disease trajectory. In addition, we combined analyses in liver biopsies and a xenograft mouse model with mechanistic experiments in cultured hepatoma cell lines to characterize the role of human STK25 in the pathogenesis of HCC.

## Results

### Hepatic STK25 Expression Positively Correlates With the Incidence and Severity of Human HCC

**Figure 1:**
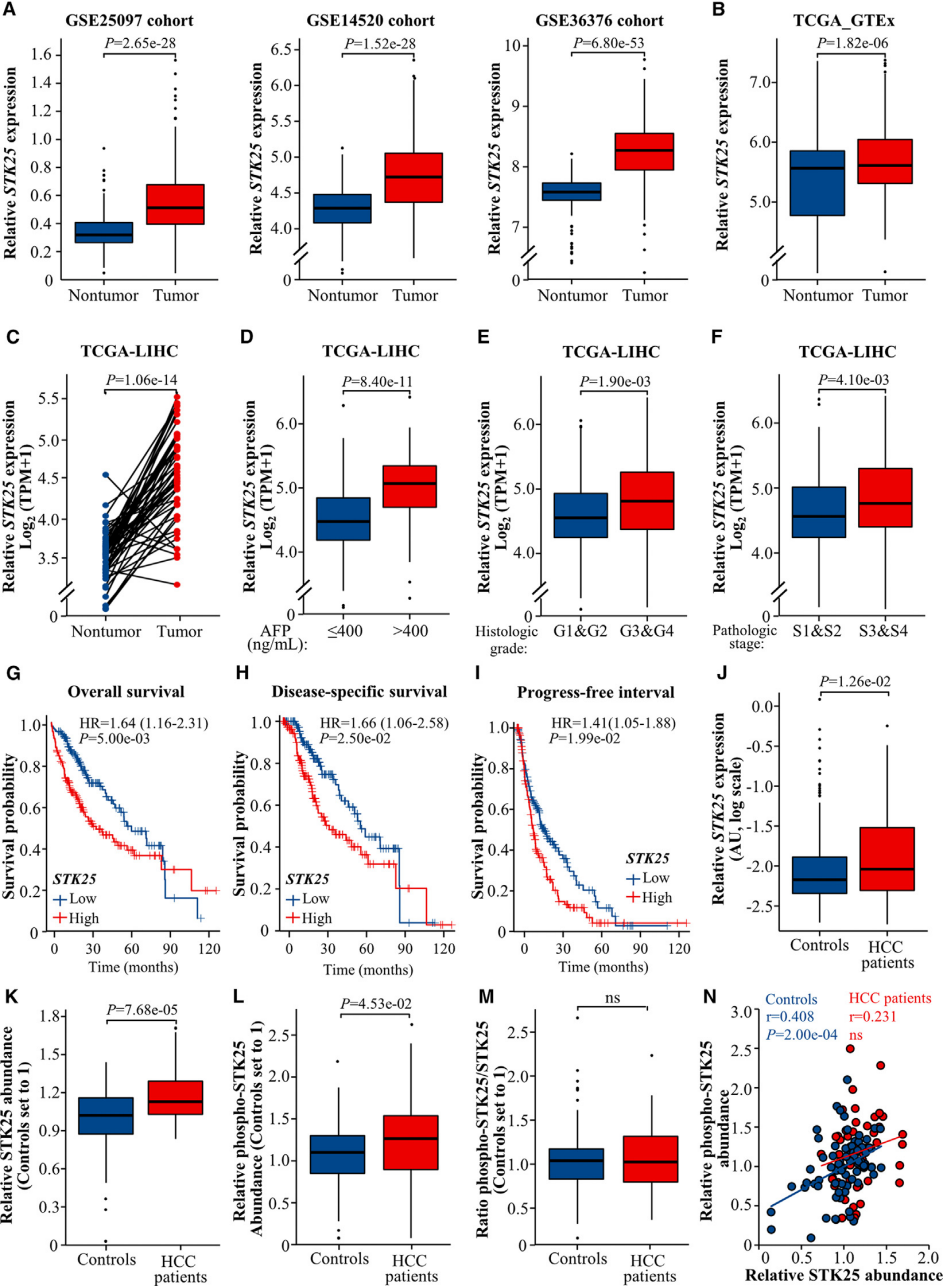
**Hepatic STK25 abundance correlates positively with the incidence and severity of human HCC.** (*A–B*) *STK25* mRNA expression in human HCC tumors vs nontumor liver tissues obtained from the GSE25097, GSE14520, GSE36376 (*A*), and combined TCGA and GTEx (*B*) datasets. (*C*) *STK25* mRNA levels in the subset of paired HCC tumors and adjacent nontumor samples. (*D–F*) *STK25* mRNA expression in individuals with low vs high levels of serum AFP (*D*), histologic HCC grade (*E*), and pathologic HCC stage (*F*). (*G–I*) Kaplan-Meier curves for overall (*G*) and disease-specific (*H*) survival, as well as progress-free interval (*I*) in patients with HCC with low vs high *STK25* levels. For (*C–I*), data are acquired from the TCGA-LIHC dataset. (*J*) Hepatic expression of *STK25* mRNA in an independent cohort of MASH-driven patients with HCC and matched controls recruited at the University Hospital of Tübingen. (*K–N*) Hepatic protein abundance of total (*K*) and phospho-STK25 (*L*) measured by PLA, the phospho-STK25/STK25 ratio (*M*), and the correlation between phospho-STK25 and STK25 (*N*) in subjects recruited at the University Hospital of Tübingen. For (*A–B, D–F, J–M*), the box plots show the median (*line in a box*), first-to-third quartiles (*boxes*), 1.5× the interquartile range (*whiskers*), and outliers (*dots*). AU, arbitrary units; HR, hazard ratio; ns, non-significant; TPM, transcripts per kilobase million.

To investigate the potential role of STK25 in the incidence of HCC, we analyzed *STK25* mRNA abundance in human HCC tumors and nontumor liver tissues by querying the microarray datasets of 3 large cohorts from the Gene Expression Omnibus (GEO) database (GSE25097: N = 268 for tumor and N = 243 for nontumor; GSE14520: N = 225 for tumor and N = 220 for nontumor; GSE36376: N = 240 for tumor and N = 193 for nontumor) and the whole RNA sequencing (RNA-seq) data combing the Cancer Genome Atlas (TCGA) and the Genotype-Tissue Expression Portal (GTEx) (N = 371 for tumor and N = 160 for nontumor). We observed a robust increase in the gene expression of STK25 in HCC tissues vs nontumor controls (Figure 1*A-B*). Moreover, in the subset of 50 paired samples from the TCGA database, *STK25* levels were higher in HCC tumors compared with the adjacent nontumor tissues (Figure 1*C*).

To further examine the possible role of STK25 in the progression of HCC, we evaluated the correlation between hepatic *STK25* mRNA levels and the clinicopathologic features of patients with HCC sourced from the TCGA database. We found that increased *STK25* expression in the livers of individuals with HCC was associated with the upregulated concentration of serum α-fetoprotein (AFP; a biomarker of a cancer-permissive tissue milieu) (Figure 1*D*) as well as advanced histologic grade (Figure 1*E*) and pathologic stage of the tumor (Figure 1*F*). Furthermore, prognostic analysis revealed that subjects with HCC with a relatively high *STK25* mRNA abundance experienced significantly shorter overall survival (Figure 1*G*), disease-specific survival (Figure 1*H*), and progress-free interval (Figure 1*I*) compared with those with a low *STK25* level.

**Figure 2:**
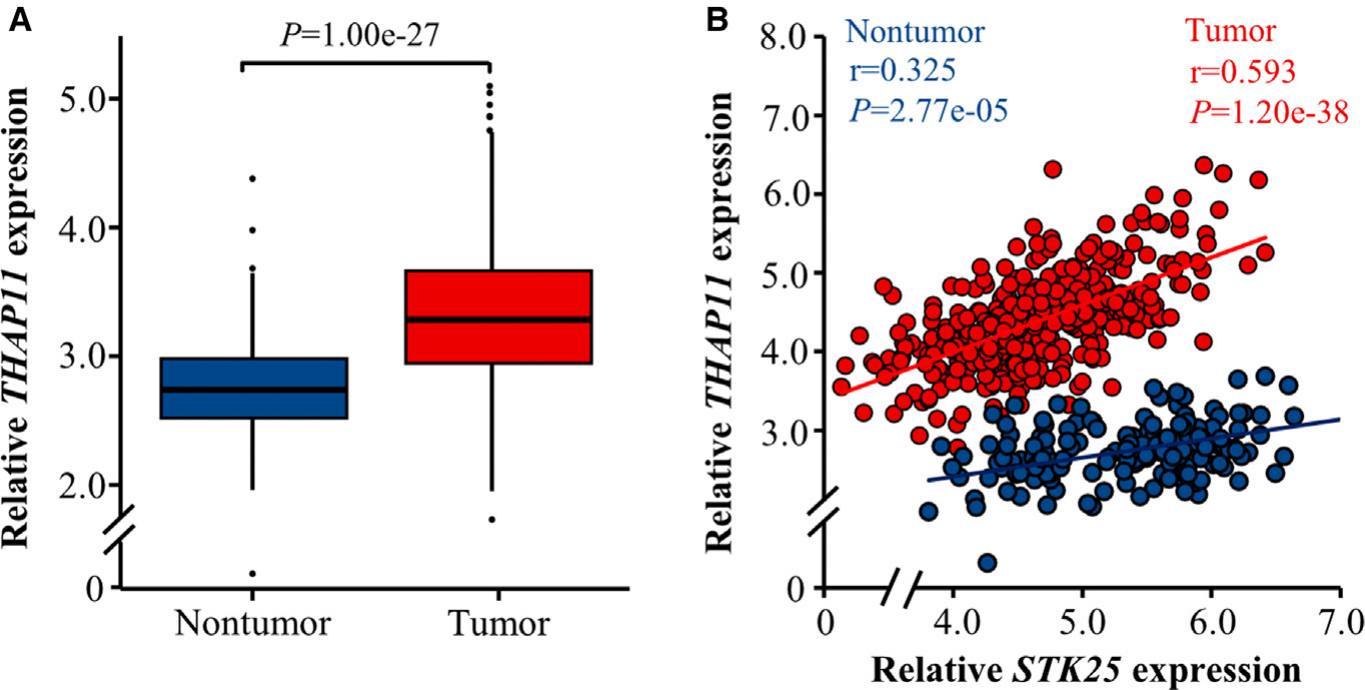
**Analysis of *THAP11* gene expression (*A*) and its association with *STK25* mRNA abundance (*B*) in human HCC tumors vs nontumor liver tissues obtained from the combined TCGA and GTEx datasets.** For (*A*), the box plots show the median (*line in a box*), first-to-third quartiles (*boxes*), 1.5× the interquartile range (*whiskers*), and outliers (*dots*).

To elucidate the potential mechanisms underlying the up-regulation of *STK25* gene expression in HCC, we performed *in silico* analysis of the promoter region (−2000 bp upstream of the first exon) to identify candidate upstream transcription factors. Utilizing the UCSC Genome Browser^19^ and the JASPAR database,^20^ a THAP11 binding site (5′-TGCCGGGACTTGTAGTCCA-3′) located at −884 to −866 bp upstream of *STK25*’s first exon was predicted. Interestingly, we also found increased *THAP11* levels in HCC tissues vs nontumor controls in the combined TCGA and GTEx databases (Figure 2*A*), and we further detected a positive correlation between hepatic *THAP11* and *STK25* mRNA expression in these datasets (Figure 2*B*). THAP11 is the most recently described member of the Thanatos-associated protein domain-containing family of atypical zinc finger transcription factors, whose transcriptional regulatory properties and roles in normal and aberrant cellular processes remain largely unexplored. Notably, altered THAP11 abundance has previously been reported in different types of cancer, with elevated expression observed in human breast and colon cancers, and decreased expression in gastric cancer, suggesting a potential role in tumorigenesis.^21^, ^22^, ^23^, ^24^, ^25^

We next complemented the bioinformatic assessment of *STK25* expression in publicly available datasets with an analysis of *STK25* mRNA abundance in the liver biopsies from an independent cohort of patients with HCC (N= 48) and control subjects (N = 214) recruited at the University Hospital of Tübingen (Tübingen, Germany) using reverse transcription quantitative polymerase chain reaction (RT-qPCR). Interestingly, also in this population, *STK25* mRNA levels were higher in HCC-bearing compared with healthy liver tissue (Figure 1*J*). Of note, while the etiology of HCC in the online databases remained unspecified, we excluded here individuals with hepatitis B and C virus infection to limit our analyses to HCC with a metabolic origin. Patients with HCC consistently displayed elevated fasting blood glucose concentration and enhanced hepatic abundance of MASH markers *TGFB1* and *COL1A1* in relation to control subjects (Table 1).

**Table 1:** Characteristics of the Study Cohort Recruited at the University Hospital of Tübingen, who Were Used for mRNA Analysis Presented in Figure 1*J*

Characteristics	Patients with HCC (N= 48)	Control subjects (N= 214)	*P*
Age, *years*	67 ± 1.7	62 ± 0.8	b
Fasting plasma glucose, *mg/dL*	114 ± 6.1	95 ± 2.6 (available in a subgroup of 92 subjects)	b
BMI, *kg/m*^*2*^	25.8 ± 0.6	25.4 ± 0.3	ns
Hepatic *TGFB1*, *arbitrary units*	0.09 ± 0.01	0.06 ± 0.01	a
Hepatic *COL1A1*, *arbitrary units*	0.37 ± 0.05	0.32 ± 0.03	b

BMI, body mass index; HCC, hepatocellular carcinoma; ns, not significant.

Data are mean ± standard error of the mean.

a *P* < .05.

b *P* < .01.

We extended our analyses by quantifying protein abundance of total and phosphorylated STK25 (phospho-STK25 [Thr^174^]; active form) by proximity ligation assay (PLA) in the liver biopsies from a subset of the study population recruited at the University Hospital of Tübingen (n = 47 for patients with HCC and n = 82 for control subjects) (see Table 2 for clinical characteristics). Both STK25 and phospho-STK25 protein levels were higher in individuals with HCC vs nontumor controls, but there was no change in the phospho-STK25/STK25 ratio (Figure 1*K–M*). Notably, we detected a significant positive correlation between phospho-STK25 and STK25 quantities in the liver biopsies obtained from controls but not in those collected from patients with HCC (Figure 1*N*), suggesting that the regulation of STK25 activity may be different in HCC-bearing compared with healthy liver tissue. It should be emphasized that individuals with HCC were slightly older than the subjects in the control groups in both the entire study panel and in the subcohort used for PLA. However, the age, fasting blood glucose, or body mass index (BMI) showed no association with the mRNA or protein expression of STK25, and the difference between the groups was independent of age, blood glucose concentration, and BMI as analyzed by a multivariate model (data not shown).

**Table 2:** Characteristics of the Study Cohort Recruited at the University Hospital of Tübingen, who Were Used for Hepatic Protein Analysis Presented in Figure 1*K–N*

Characteristics	Patients with HCC (n = 47)	Control subjects (n = 82)	*P*
Age, *years*	67 ± 1.5	62 ± 1.2	a
Fasting plasma glucose, *mg/dL*	110 ± 6.5 (available in a subgroup of 16 subjects)	100 ± 4.3 (available in a subgroup of 37 subjects)	ns
BMI, *kg/m*^*2*^	25.8 ± 0.6	25.2 ± 0.5	ns
Hepatic *TGFB1*, *arbitrary units*	0.09 ± 0.01	0.05 ± 0.01	b
Hepatic *COL1A1*, *arbitrary units*	0.36 ± 0.05	0.24 ± 0.04	b

BMI, body mass index; HCC, hepatocellular carcinoma; ns, not significant.

Data are mean ± standard error of the mean.

a *P* < .05.

b *P* < .001.

### Knockout of STK25 Blocks Hepatocarcinogenesis in a Subcutaneous Xenograft Mouse Model

**Figure 3:**
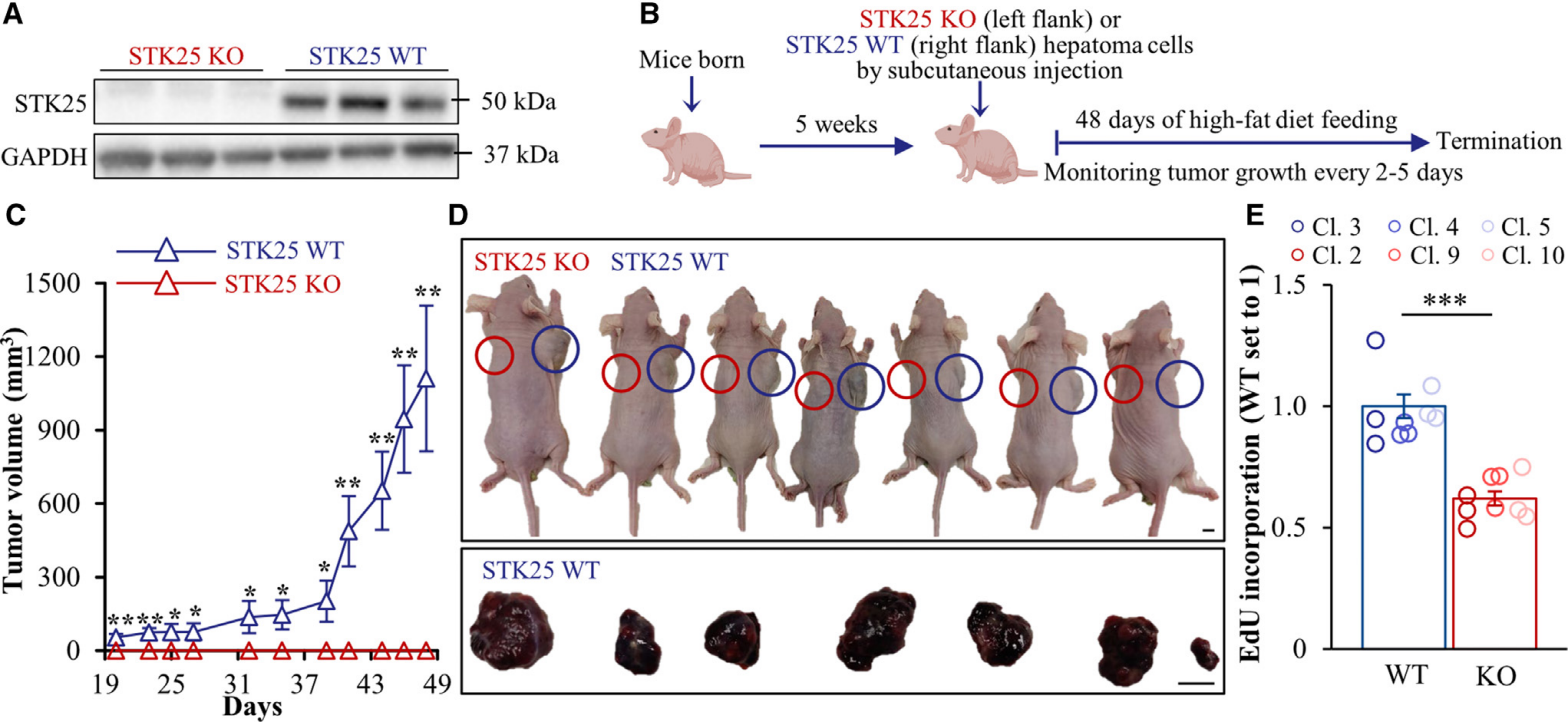
**STK25 knockout blocks hepatocarcinogenesis in a subcutaneous xenograft mouse model.** (*A*) STK25 protein abundance in STK25-depleted and wild-type HepG2 cells was assessed by Western blot. Representative Western blots are shown with glyceraldehyde-3-phosphate dehydrogenase (GAPDH) used as a loading control. (*B*) Schematic presentation of the experimental design. (*C*) Xenograft tumor growth measured over time. (*D*) Images of tumor-bearing mice (*upper*) and xenograft tumors (*lower*) at the day of termination. No visible tumors were detected in the left flanks of mice inoculated with STK25-depleted HepG2 cells. Scale bar: 5 mm. (*E*) Cell proliferation of STK25 knockout vs wild-type HepG2 cells analyzed by EdU incorporation assay. Data are mean ± SEM from 7 mice (*C*) or 3 clones (*E*) per group. Cl., clone; KO, knockout; WT, wild-type. ∗*P* < .05; ∗∗*P* < .01; ∗∗∗*P* < .001.

Having observed that STK25 mRNA and protein levels are increased in human HCC, we next investigated the impact of STK25 on hepatocarcinogenesis in a subcutaneous xenograft model in mice. We generated stable knockout of STK25 in HepG2 (human hepatoblastoma cells) utilizing CRISPR/Cas9 technology and injected these cells subcutaneously into the flanks of high-fat diet-fed BALB/c nude mice (Figure 3*A–B*). Remarkably, the deletion of STK25 completely blocked in vivo tumor formation and growth ability of HepG2 cells, whereas all mice inoculated with wild-type HepG2 cells developed tumors of variable size (Figure 3*C–D*). Consistent with these in vivo findings, we detected reduced proliferation of STK25-depleted vs wild-type HepG2 clones cultured in vitro (Figure 3*E*), whereas cell viability was unaffected by STK25 knockout (viability of 98%–100% was observed in both STK25 knockout and wild-type HepG2 cells, as determined by Trypan Blue staining, both before and after subcutaneous injections).

### Stk25-Targeting ASO Therapy Hinders the Development and Progression of MASH-Related HCC in a Mouse Model

**Figure 4:**
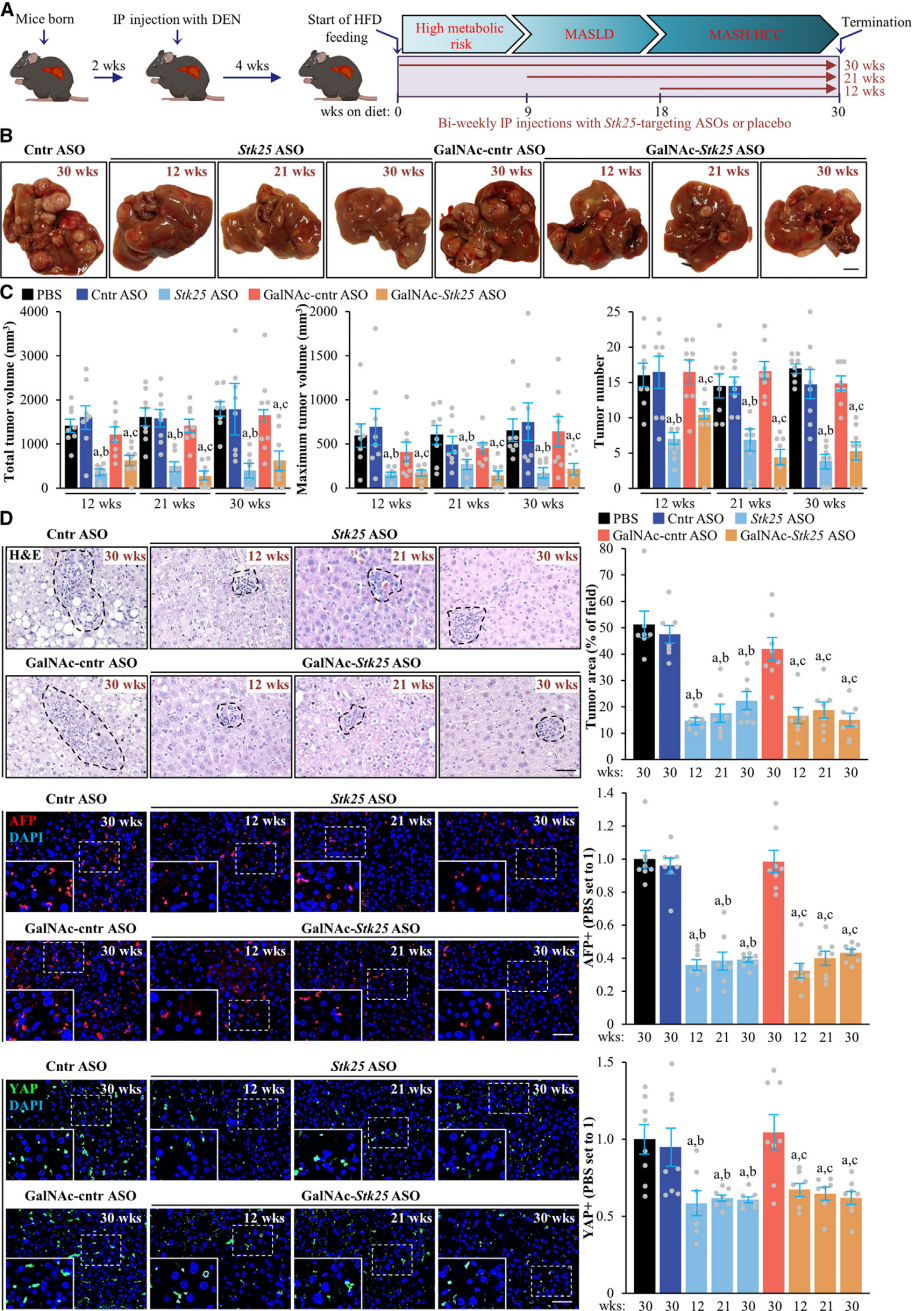
**Administration of *Stk25*-targeting ASOs suppresses the development and progression of MASH-related HCC in a mouse model.** (*A*) Schematic presentation of the experimental design. (*B*) Representative images of whole liver. Scale bar: 5 mm. (*C*) Quantification of volume and number of the visible tumors on the liver surface. (*D*) Representative liver sections stained with H&E or processed for immunofluorescence with anti-AFP (*red*) or anti-YAP (*green*) antibodies; nuclei stained with DAPI (*blue*). Quantification of the tumor area and immunofluorescence staining. Scale bar: 50 μm. Numbers under each bar graph and in the upper right corner of each image indicate the weeks of treatment with *Stk25*-targeting ASOs or placebo. Data are mean ± SEM from 7 to 8 mice per group. Cntr, control; HFD, high-fat diet; IP, intraperitoneal; wks, weeks. ^a^*P* < .05 for *Stk25*-targeting ASOs vs PBS; ^b^*P* < .05 for *Stk25* ASO vs Cntr ASO; ^c^*P* < .05 for GalNAc-*Stk25* ASO vs GalNAc-cntr ASO.

**Figure 5:**
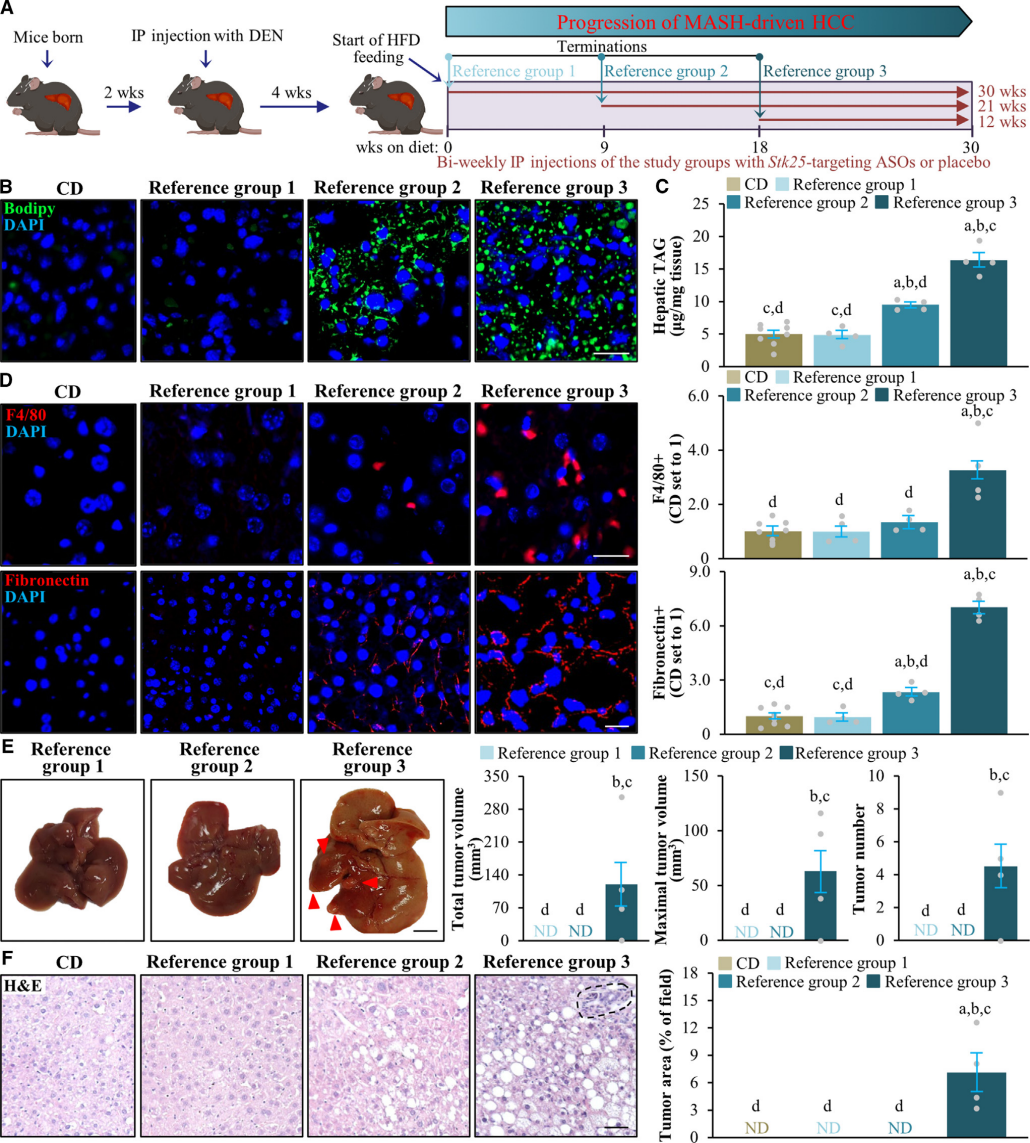
**ASO treatment was initiated in mice at the different phases of the disease trajectory.** (*A*) Schematic presentation of the termination points for reference groups. Chow-fed mice were killed 34 weeks after DEN injection. (*B*) Representative liver sections stained with Bodipy (*green*); nuclei stained with DAPI (*blue*). Scale bar: 50 μm. (*C*) Hepatic TAG content measured by colorimetric assay. (*D*) Representative liver sections processed for immunofluorescence with anti-F4/80 (*red*) or anti-fibronectin (*red*) antibodies; nuclei stained with DAPI (*blue*). Quantification of the staining. Scale bar: 50 μm. (*E*) Representative images of whole liver. Quantification of volume and number of the visible tumors on the liver surface. *Arrowheads* indicate visible tumors. Scale bar: 5 mm. (*F*) Representative liver sections stained with H&E. Quantification of tumor area. Scale bar: 50 μm. Data are mean ± SEM from 4 (reference groups) or 8 (CD group) mice per group. CD, chow-diet; HFD, high-fat diet; IP, intraperitoneal; ND, non-detectable; wks, weeks. ^a^*P* < .05 vs CD; ^b^*P* < .05 vs reference group 1; ^c^*P* < .05 vs reference group 2; ^d^*P* < .05 vs reference group 3.

We next sought to demonstrate the in vivo proof-of-principle for the efficacy of pharmacologic STK25 inhibitors in the prevention and/or treatment of MASH-associated HCC. To achieve this, we used a mouse model where HCC was induced in the context of MASH by a single injection of chemical procarcinogen diethylnitrosamine (DEN) in combination with a high-fat diet challenge for 30 weeks. Hepatocyte-specific N-acetylgalactosamine (GalNAc)-conjugated *Stk25* ASO and the broadly acting parent unconjugated *Stk25* ASO (complementary to the identical 16-nucleotide intronic region of the mouse *Stk25* gene) were administered by bi-weekly intraperitoneal injections. We also included the reference groups of mice that received the control ASOs (non-targeting ASOs of the same length and chemistry as GalNAc-*Stk25* ASO or *Stk25* ASO) and the phosphate-buffered saline (PBS)-treated groups. The ASO administration was initiated in 3 cohorts of mice at the different phases of the disease trajectory as follows: (1) metabolically healthy mice with normal liver fat content (group dosed for 30 weeks); (2) mice with mild MASLD (i.e., simple hepatic steatosis without extensive liver inflammation or fibrosis; group dosed for 21 weeks); or (3) mice having MASH (i.e., overt liver steatosis, inflammation, and fibrosis) with no or few HCC tumors (group dosed for 12 weeks) (see Figure 4*A* for a schematic overview of the experimental design; Figure 5).

**Figure 6:**
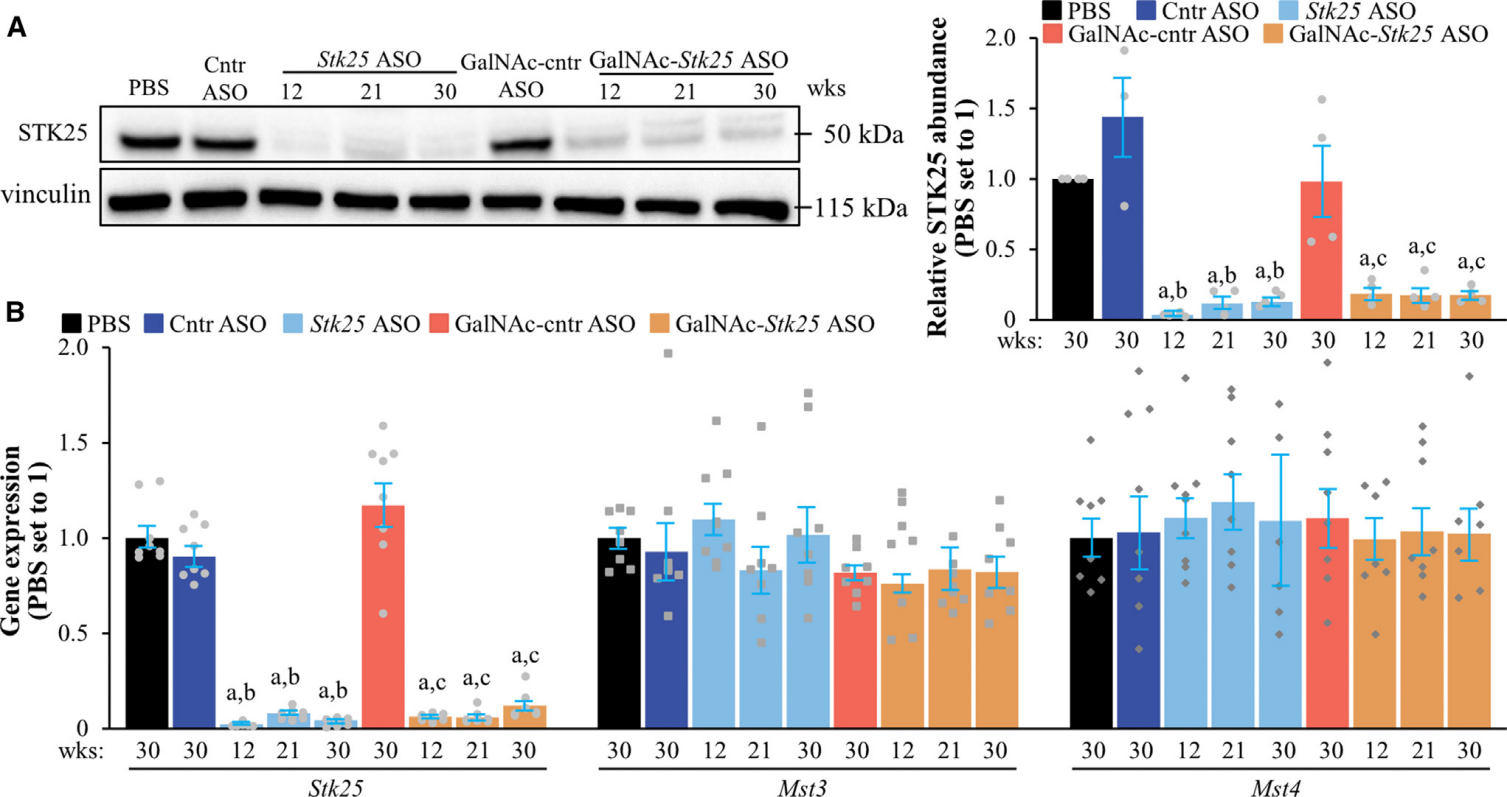
**Analysis of the abundance of STK25 and related kinases in the livers of mice with experimentally induced MASH-driven HCC.** (*A*) Liver lysates were analyzed by Western blot using antibodies specific for STK25. Protein levels were assessed by densitometry; representative Western blots are shown with vinculin used as loading control. (*B*) Relative mRNA expression of *Stk25*, *Mst3*, and *Mst4* was determined by RT-qPCR in the liver. Numbers under each bar graph and above representative Western blots indicate the weeks of treatment with *Stk25*-targeting ASOs or placebo. Data are mean ± SEM from 4 (*A*) or 8 (*B*) mice per group. Cntr, control; wks, weeks. ^a^*P* < .05 for *Stk25-*targeting ASOs vs PBS; ^b^*P* < .05 for *Stk25* ASO vs Cntr ASO; ^c^*P* < .05 for GalNAc-*Stk25* ASO vs GalNAc-cntr ASO.

The applied doses of *Stk25* ASO and GalNAc-*Stk25* ASO (50 and 12.5 mg/kg/week, respectively) were selected based on our previous reports demonstrating the suppression of STK25 expression in mouse liver by about 90%,^14^^,^^17^ which was also confirmed in this study (Figure 6). Notably, the treatment with *Stk25*-targeting ASOs did not affect the hepatic mRNA levels of the 2 kinases closely related to STK25—MST3 and MST4 (Figure 6*B*).

**Figure 7:**
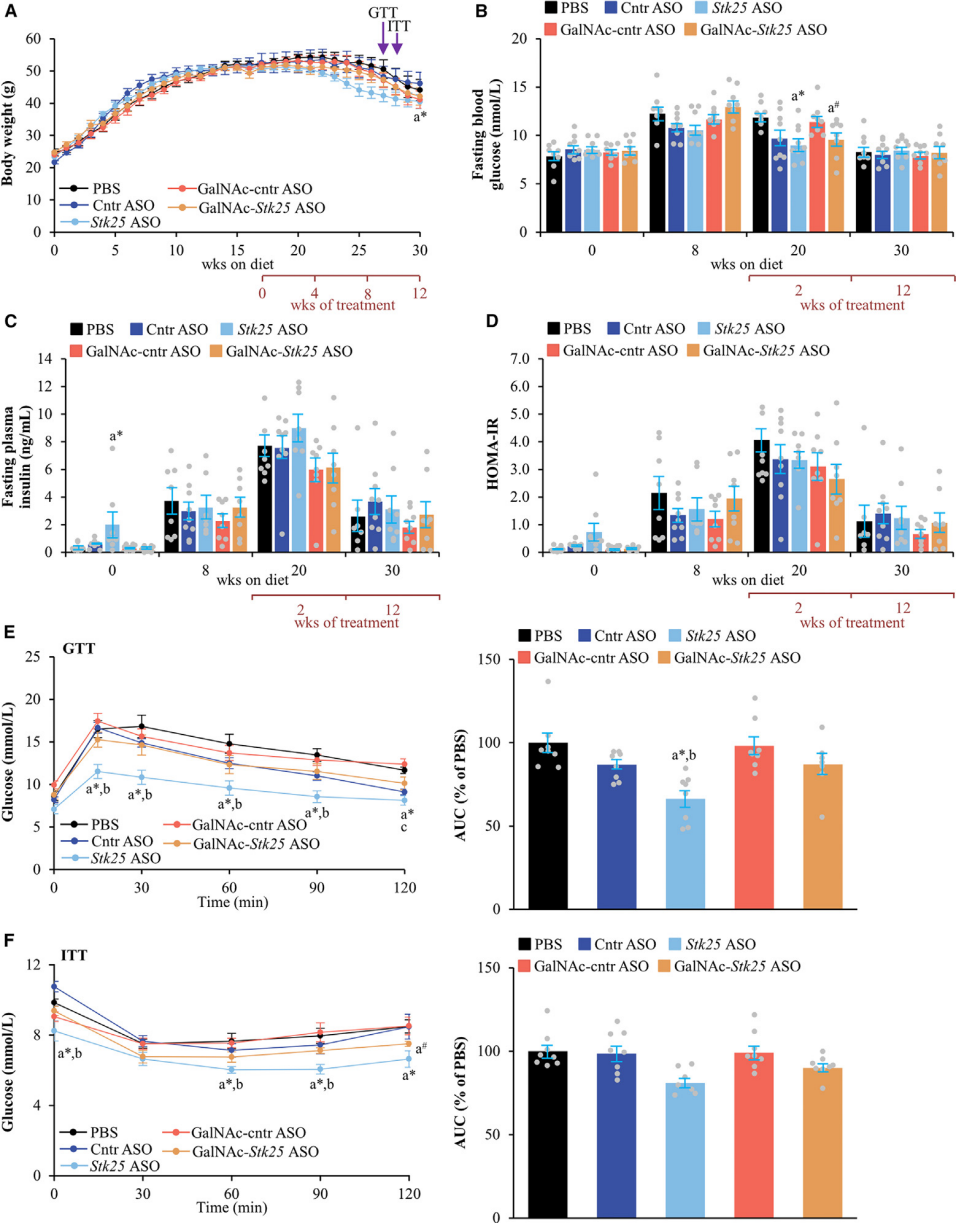
**Assessment of glucose and insulin homeostasis in mice with experimentally induced MASH-driven HCC treated with *Stk25-*targeting ASOs or placebo for 12 weeks.** (*A*) Body weight curves. (*B*–*C*) Fasting circulating levels of glucose (*B*) and insulin (*C*). (*D*) HOMA-IR calculated using the equation (fasting glucose [mg/dL] × fasting insulin [ng/mL])/405. (*E*–*F*) Intraperitoneal GTT (*E*) and ITT (*F*) performed after 9 and 10 weeks of treatment with *Stk25*-targeting ASOs or placebo, respectively. The area under the glucose curve in each test. Data are mean ± SEM from 7 to 9 mice per group. AUC, area under the curve; Cntr, control; wks, weeks. ^a^∗*P* < .05 for *Stk25* ASO vs PBS; ^a#^*P* < .05 for GalNAc-*Stk25* ASO vs PBS; ^b^*P* < .05 for *Stk25* ASO vs Cntr ASO; ^c^*P* < .05 for GalNAc-*Stk25* ASO vs GalNAc-cntr ASO.

**Figure 8:**
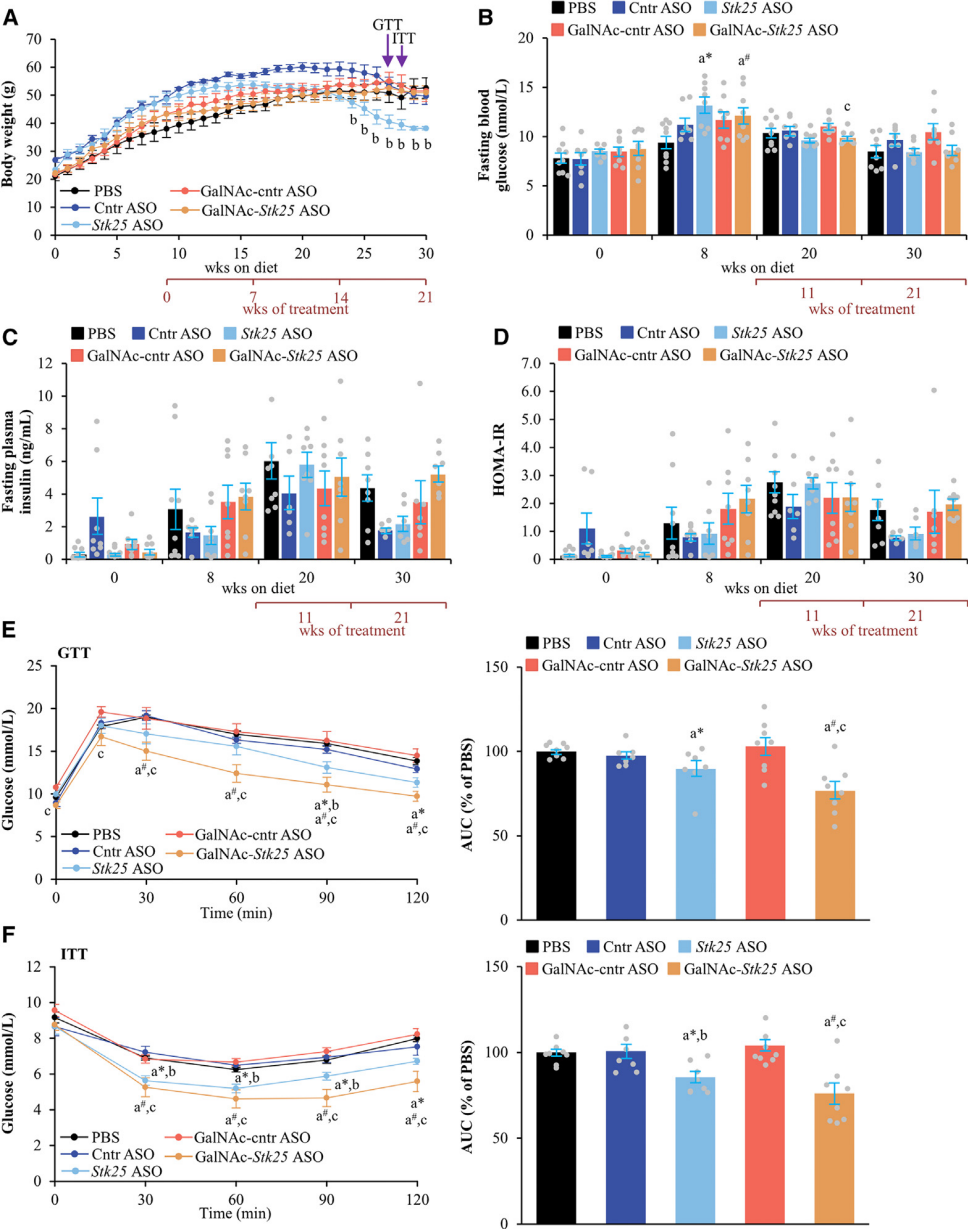
**Analysis of glucose and insulin homeostasis in mice with experimentally induced MASH-driven HCC treated with *Stk25-*targeting ASOs or placebo for 21 weeks.** (*A*) Body weight curves. (*B*–*C*) Fasting circulating levels of glucose (*B*) and insulin (*C*). (*D*) HOMA-IR calculated using the equation (fasting glucose [mg/dL] × fasting insulin [ng/mL])/405. (*E*–*F*) Intraperitoneal GTT (*E*) and ITT (*F*) performed after 18 and 19 weeks of treatment with *Stk25*-targeting ASOs or placebo, respectively. The area under the glucose curve in each test. Data are mean ± SEM from 5 to 9 mice per group. AUC, area under the curve; Cntr, control; wks, weeks. ^a^∗*P* < .05 for *Stk25* ASO vs PBS; ^a#^*P* < .05 for GalNAc-*Stk25* ASO vs PBS; ^b^*P* < .05 for *Stk25* ASO vs Cntr ASO; ^c^*P* < .05 for GalNAc-*Stk25* ASO vs GalNAc-cntr ASO.

**Figure 9:**
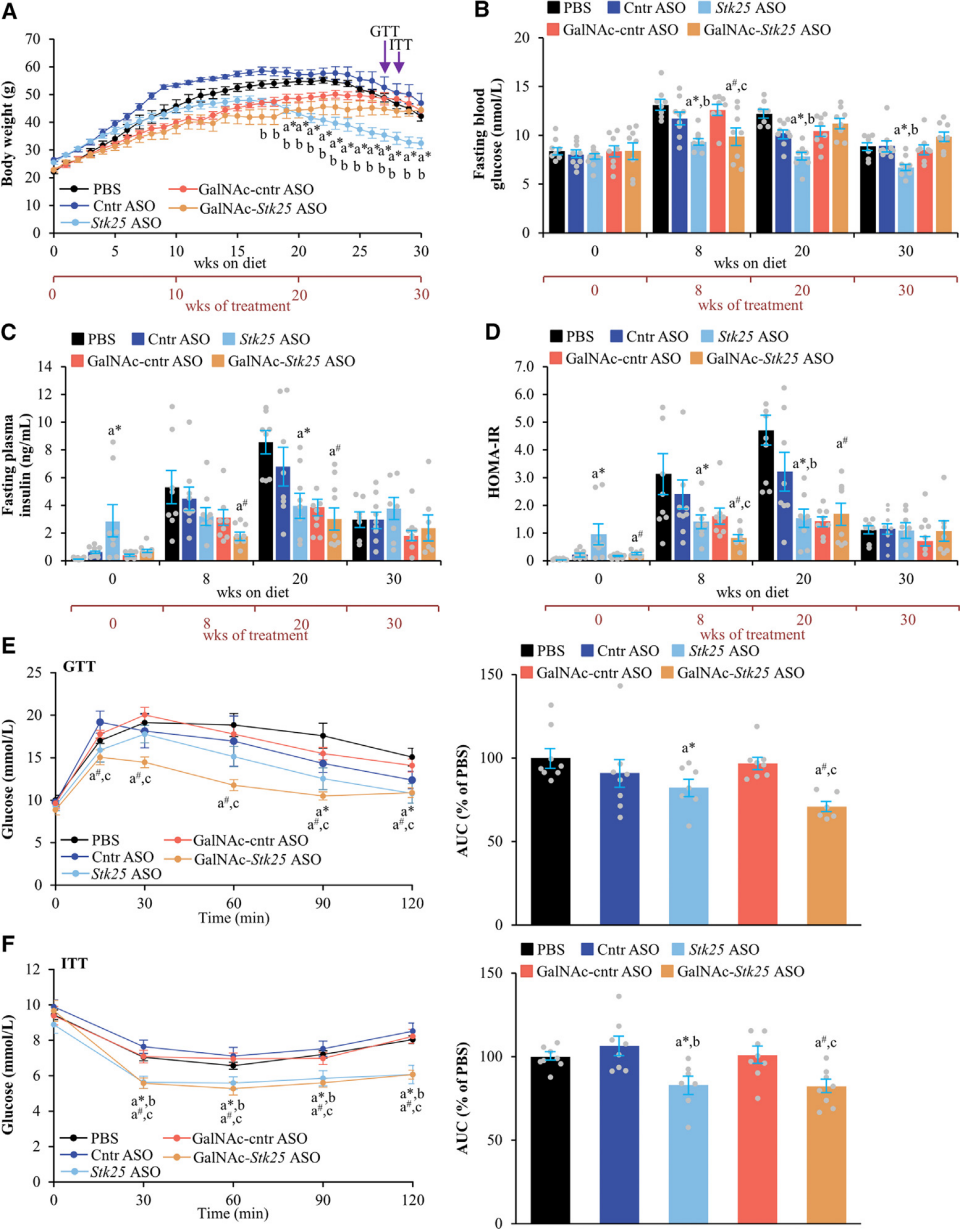
**Assessment of glucose and insulin homeostasis in mice with experimentally induced MASH-driven HCC treated with *Stk25-*targeting ASOs or placebo for 30 weeks.** (*A*) Body weight curves. (*B*–*C*) Fasting circulating levels of glucose (*B*) and insulin (*C*). (*D*) HOMA-IR calculated using the equation (fasting glucose [mg/dL] × fasting insulin [ng/mL])/405. (*E*–*F*) Intraperitoneal GTT (*E*) and ITT (*F*) performed after 27 and 28 weeks of treatment with *Stk25*-targeting ASOs or placebo, respectively. The area under the glucose curve in each test. Data are mean ± SEM from 6 to 9 mice per group. AUC, area under the curve; Cntr, control; wks, weeks. ^a^∗*P* < .05 for *Stk25* ASO vs PBS; ^a#^*P* < .05 for GalNAc-*Stk25* ASO vs PBS; ^b^*P* < .05 for *Stk25* ASO vs Cntr ASO; ^c^*P* < .05 for GalNAc-*Stk25* ASO vs GalNAc-cntr ASO.

We first examined the effect of ASO administration on measures of whole-body metabolic physiology. Long-term administration of *Stk25* ASO modestly reduced body weight, whereas comparable weights were observed in mice receiving GalNAc-*Stk25* ASO vs placebo throughout the experiment (Figures 7*A*, 8*A*, 9*A*). Suppression of STK25 abundance did not lead to consistent alterations in fasting blood glucose, plasma insulin, or the homeostasis model assessment score of insulin resistance (HOMA-IR), measured at several time points during the study (Figures 7*B–D*, 8*B–D*, 9*B–D*). Of note, improved glucose tolerance and insulin sensitivity assessed by glucose tolerance test (GTT) and insulin tolerance test (ITT), respectively, were detected in all groups of mice that received *Stk25-*targeting ASOs for 21 or 30 weeks when compared with the control groups. In the cohort dosed for 12 weeks, only glucose tolerance was improved in *Stk25* ASO- vs placebo-treated mice (Figures 7*E–F*, 8*E–F*, 9*E–F*).

**Figure 10:**
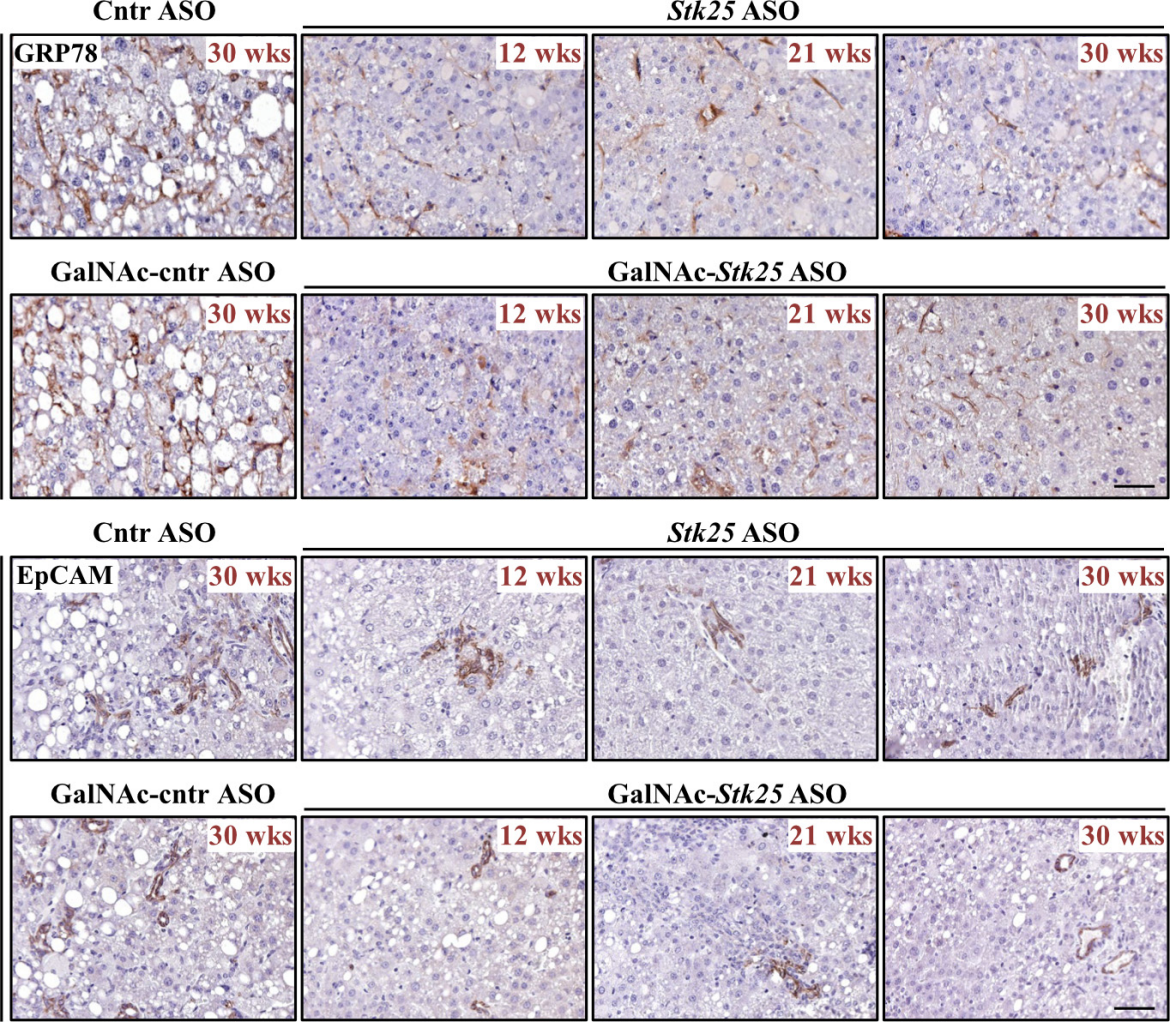
**Analysis of hepatic GRP78 and EpCAM abundance in mice with experimentally induced MASH-driven HCC.** Representative liver sections processed for immunohistochemistry with anti-GRP78 (*brown*) or anti-EpCAM (*brown*) antibodies; counterstaining with hematoxylin. Scale bar: 50 μm. Numbers in the upper right corner of each image indicate the weeks of treatment with *Stk25*-targeting ASOs or placebo. Cntr, control; wks, weeks.

Next, we quantified tumor burden in these cohorts. Interestingly, all mice receiving placebo developed numerous macroscopic nodules of variable size on the liver surface by study termination, whereas about 20% of mice dosed with *Stk25*-targeting ASOs did not display any tumors upon gross inspection of the liver (Figure 4*B–C*). The total and maximum volume, as well as the number of visible HCC tumors, were also significantly lower in mice treated with *Stk25* ASO or GalNAc-*Stk25* ASO (Figure 4*B–C*). These macroscopic observations were supported by histologic assessment. Specifically, we observed reduced tumor area in the hematoxylin and eosin (H&E)-stained liver sections from mice receiving *Stk25-*targeting ASOs vs placebo, which was accompanied by a decreased hepatic immunolabeling for AFP, Yes-associated protein (YAP), glucose regulatory protein 78 (GRP78), and epithelial cell adhesion molecule (EpCAM), whose abundance is known to positively correlate with the tumor grade and dismal prognosis in patients with HCC^3^^,^^26^, ^27^, ^28^ (Figures 4*D*, 10)

**Figure 11:**
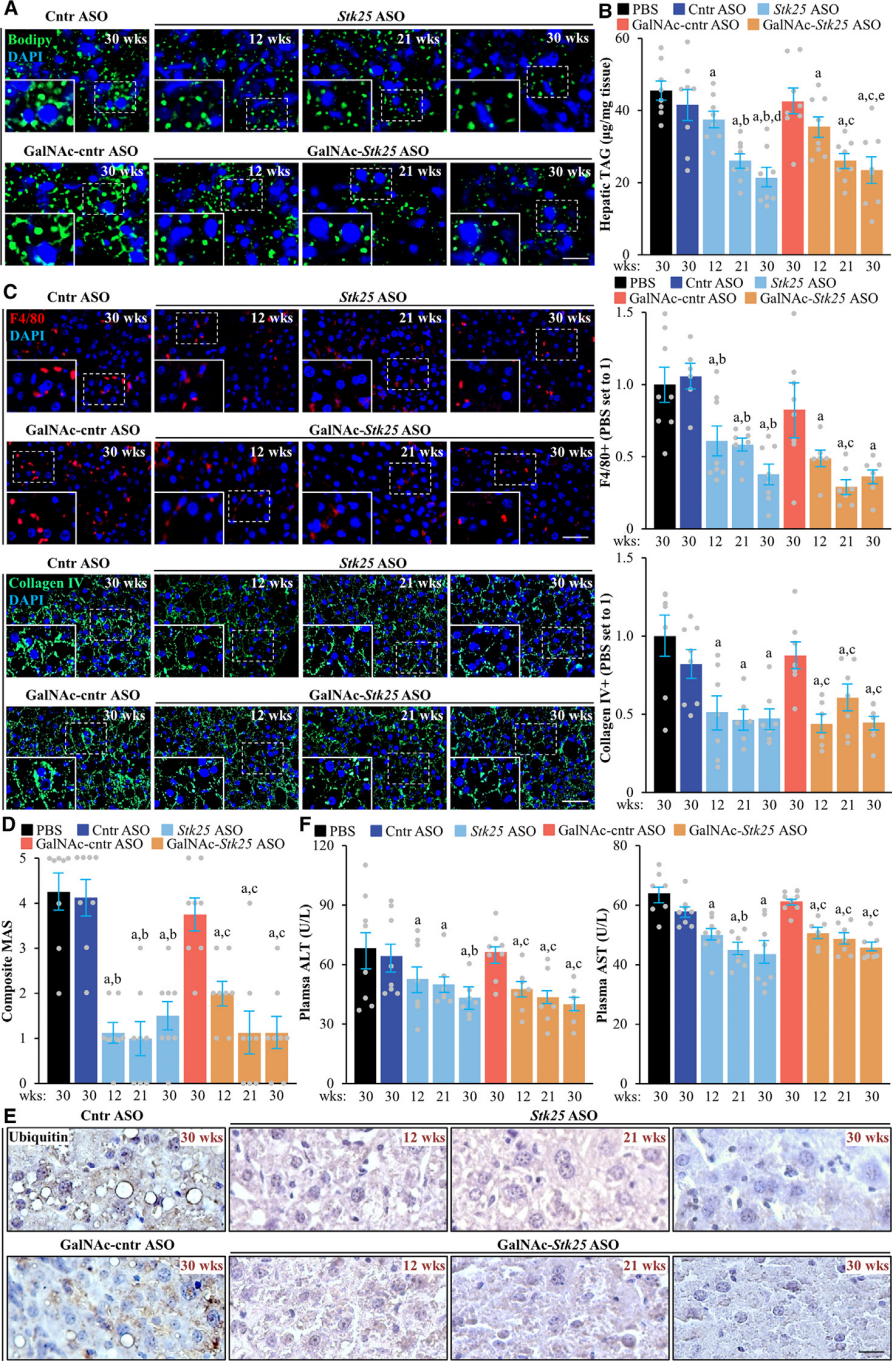
**Mice treated with *Stk25*-targeting ASOs display a marked suppression of MASH severity.** (*A*) Representative liver sections stained with Bodipy 493/503 (*green*); nuclei stained with DAPI (*blue*). Scale bar: 25 μm. (*B*) Hepatic TAG content measured by colorimetric assay. (*C*) Representative liver sections processed for immunofluorescence with anti-F4/80 (*red*) or anti-collagen IV (*green*) antibodies; nuclei stained with DAPI (*blue*). Quantification of the staining. Scale bar: 50 μm. (*D*) Assessment of composite MAS in H&E-stained liver sections. (*E*) Representative liver sections processed for immunohistochemistry with anti-ubiquitin (*brown*) antibodies; counterstaining with hematoxylin. Scale bar: 25 μm. (*F*) Measurement of plasma ALT and AST concentrations. Numbers under each bar graph and in the upper right corner of each image indicate the weeks of treatment with *Stk25*-targeting ASOs or placebo. Data are mean ± SEM from 7 to 8 mice per group. Cntr, control; wks, weeks. ^a^*P* < .05 for *Stk25*-targeting ASOs vs PBS; ^b^*P* < .05 for *Stk25* ASO vs Cntr ASO; ^c^*P* < .05 for GalNAc-*Stk25* ASO vs GalNAc-cntr ASO; ^d^*P* < .05 for *Stk25* ASO for 30 weeks vs 12 weeks; ^e^*P* < .05 for GalNAc-*Stk25* ASO for 30 weeks vs 12 weeks.

**Figure 12:**
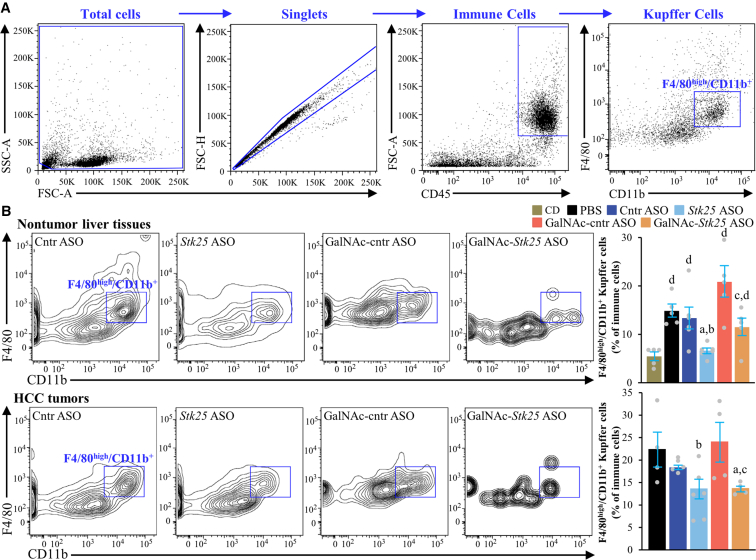
**Quantification of Kupffer cells by flow cytometry in the livers of mice with experimentally induced MASH-driven HCC treated with *Stk25*-targeting ASOs or placebo for 12 weeks.** Chow-fed mice were included for reference. (*A*) Gating strategies. F4/80^high^/CD11b^+^ subpopulation was identified as Kupffer cells. (*B*) Representative flow cytometry plots and quantification of F4/80^high^/CD11b^+^ Kupffer cells in nontumor liver tissues and HCC tumors. *Blue boxes* indicate the gates used for sorting and quantification. Data are mean ± SEM from 4 to 6 mice per group. CD, chow-diet; Cntr, control; FSC, forward scatter; SSC, side scatter. ^a^*P* < .05 for *Stk25-*targeting ASOs vs PBS; ^b^*P* < .05 for *Stk25* ASO vs Cntr ASO; ^c^*P* < .05 for GalNAc-*Stk25* ASO vs GalNAc-cntr ASO; ^d^*P* < .05 for vs CD.

**Figure 13:**
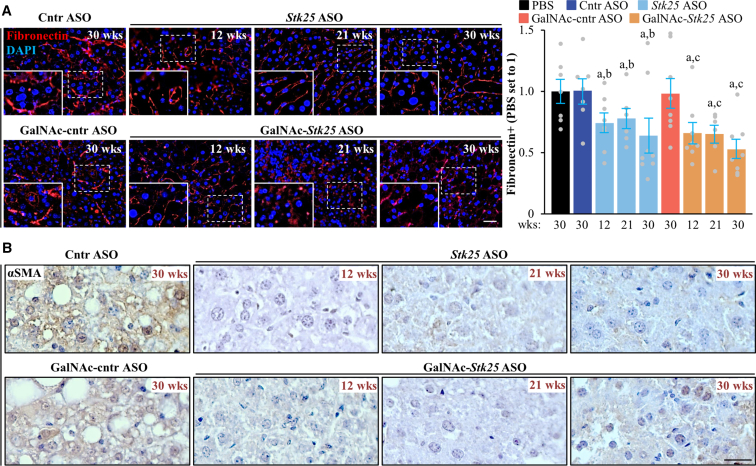
**Analysis of hepatic fibronectin and αSMA abundance in mice with experimentally induced MASH-driven HCC.** (*A*) Representative liver sections processed for immunofluorescence with anti-fibronectin (*red*) antibodies; nuclei stained with DAPI (*blue*). Quantification of the staining. Scale bar: 50 μm. (*B*) Representative liver sections processed for immunohistochemistry with anti-αSMA (*brown*) antibodies; counterstaining with hematoxylin. Scale bar: 25 μm. Numbers under bar graph and in the upper right corner of each image indicate the weeks of treatment with *Stk25-*targeting ASOs or placebo. Data are mean ± SEM from 7 to 8 mice per group. Cntr, control; wks, weeks. ^a^*P* < .05 for *Stk25*-targeting ASOs vs PBS; ^b^*P* < .05 for *Stk25* ASO vs Cntr ASO; ^c^*P* < .05 for GalNAc-*Stk25* ASO vs GalNAc-cntr ASO.

**Figure 14:**
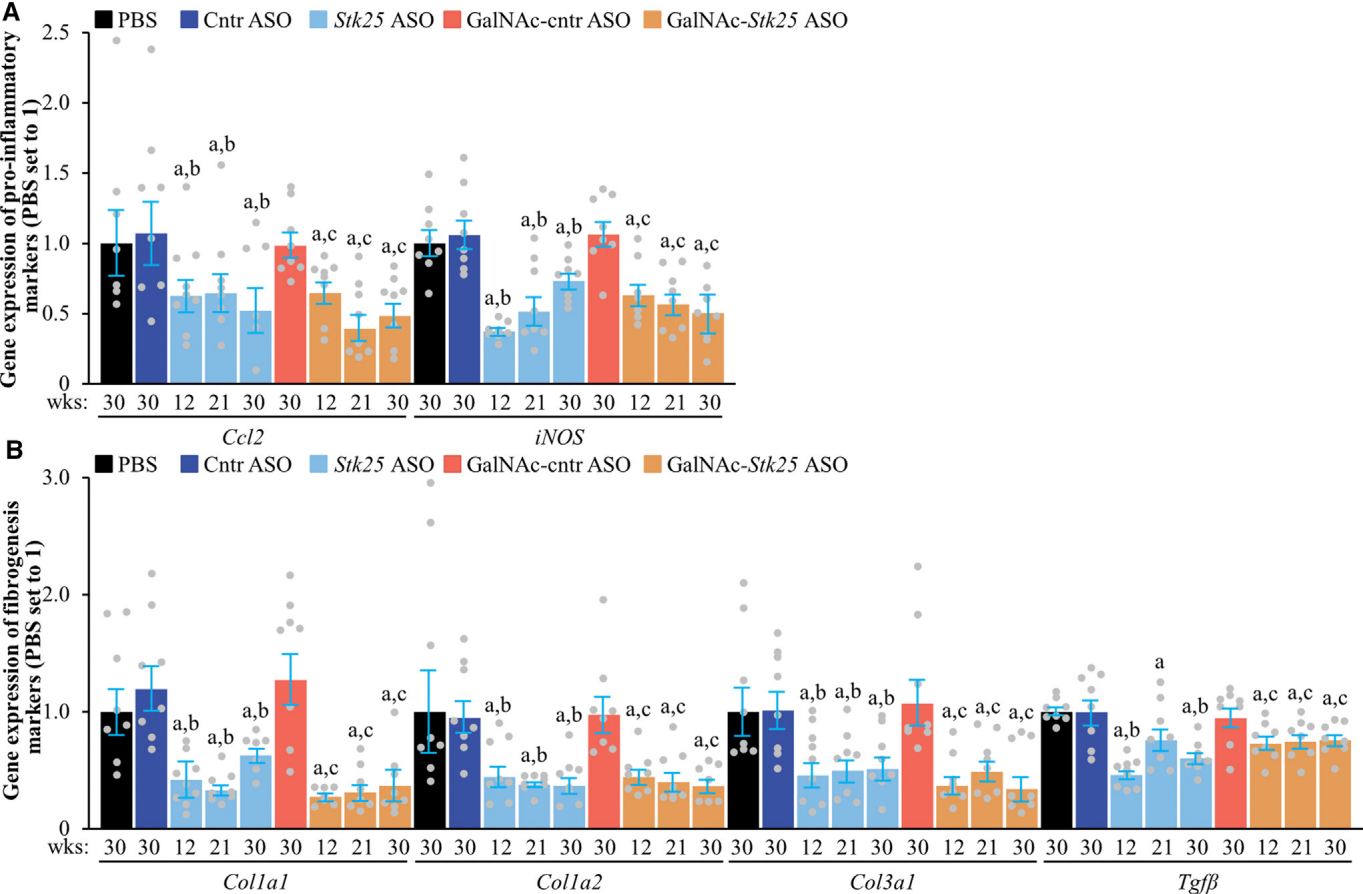
**Measurement of mRNA levels of selected markers of inflammation (*A*) and fibrogenesis (*B*) in the livers of mice with experimentally induced MASH-driven HCC by RT-qPCR.** Numbers under each bar graph indicate the weeks of treatment with *Stk25-*targeting ASOs or placebo. Data are mean ± SEM from 8 mice per group. Cntr, control; wks, weeks. ^a^*P* < .05 for *Stk25-*targeting ASOs vs PBS; ^b^*P* < .05 for *Stk25* ASO vs Cntr ASO; ^c^*P* < .05 for GalNAc-*Stk25* ASO vs GalNAc-cntr ASO.

**Figure 15:**
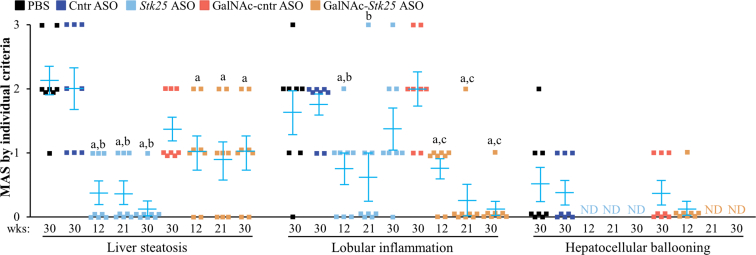
**Assessment of individual histological features of MAS in H&E-stained liver sections from mice with experimentally induced MASH-driven HCC.** Scoring was performed by an independent histopathologist based on 3 semiquantitative components: liver steatosis (0–3), lobular inflammation (0–3), and hepatocellular ballooning (0–2). Numbers under bar graph indicate the weeks of treatment with *Stk25*-targeting ASOs or placebo. Data are mean ± SEM from 8 mice per group. Cntr, control; ND, non-detectable; wks, weeks. ^a^*P* < .05 for *Stk25-*targeting ASOs vs PBS; ^b^*P* < .05 for *Stk25* ASO vs Cntr ASO; ^c^*P* < .05 for GalNAc-*Stk25* ASO vs GalNAc-cntr ASO.

In line with our previous studies,^14^^,^^17^ *Stk25* ASO- or GalNAc-*Stk25* ASO-treated mice displayed a substantial attenuation of the pathologic features of MASH as evidenced by: (1) reduced hepatic steatosis measured by histologic staining with the lipophilic dye Bodipy 493/503 and colorimetric assay for lipids (Figure 11*A–B*); (2) inhibited inflammatory infiltration in the livers determined by immunofluorescence staining for macrophage marker F4/80 and flow cytometry quantifying F4/80^high^/CD11b^+^ Kupffer cells (Figures 11*C*, 12); and (3) down-regulated hepatic fibrosis assessed by immunolabeling for collagen IV, fibronectin, and α-smooth muscle actin (αSMA; a marker for activated hepatic stellate cells [HSCs] that secrete collagen) (Figures 11*C*, 13). The mRNA expression of several markers of liver inflammation and fibrogenesis was also significantly lower in mice dosed with *Stk25*-targeting ASOs than in the control groups (Figure 14). Consistently, histopathologic evaluation performed in H&E-stained liver sections using MASLD Activity Score (MAS) following the Kleiner/Brunt criteria adapted for use in rodents^29^^,^^30^ demonstrated that suppression of STK25 levels significantly decreased the composite MAS (comprised of liver steatosis, lobular inflammation, and hepatocellular ballooning scores) (Figures 11*D*, 15). Mallory-Denk bodies (MDBs) have been reported as one of the most relevant histologic indices correlating with MASH disease activity and were recently incorporated into the expanded MAS.^31^ To this end, we found that MDBs (shown by immunohistochemical analysis for ubiquitin) were readily detected in the liver sections from placebo-treated mice but not in those from mice receiving *Stk25* ASO or GalNAc-*Stk25* ASO (Figure 11*E*). Furthermore, plasma concentrations of alanine transaminase (ALT) and aspartate aminotransferase (AST), the most widely used clinical biomarkers of MASH, were downregulated in mice dosed with *Stk25*-targeting ASOs compared with mice in the control groups (Figure 11*F*).

**Figure 16:**
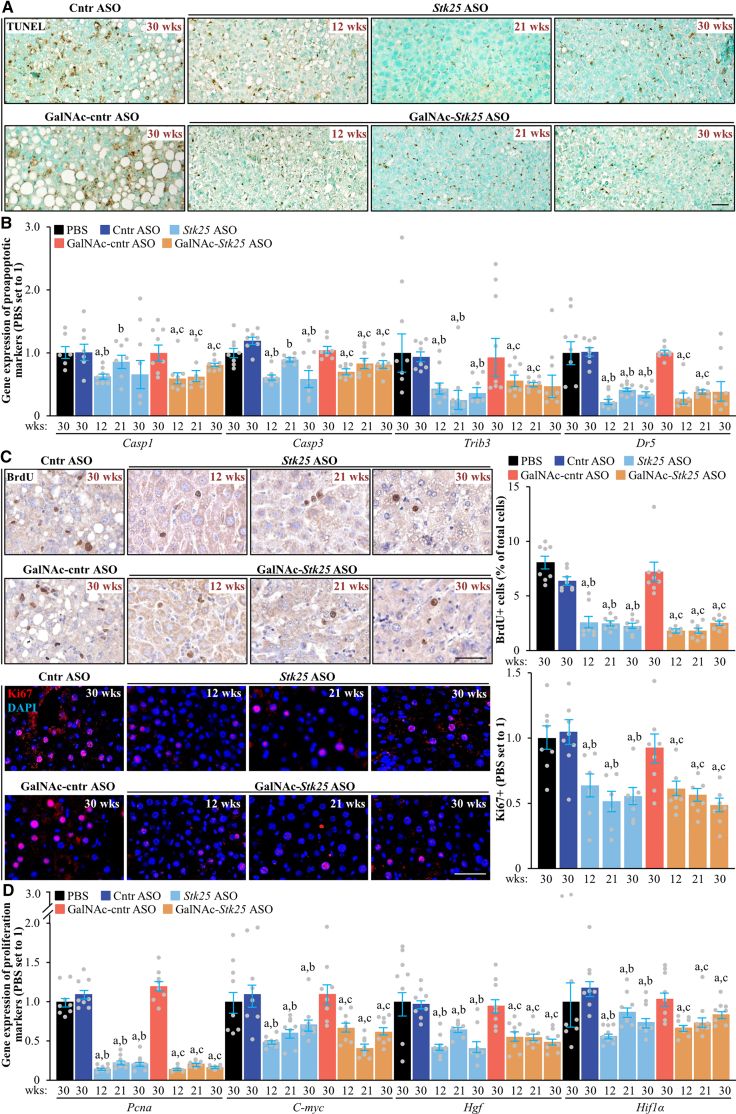
**Treatment with *Stk25*-targeting ASOs attenuates hepatocellular apoptosis and proliferation in mice with experimentally induced MASH-driven HCC.** (*A*) Representative liver sections stained with TUNEL (*brown*); counterstaining with methyl green. Scale bar: 50 μm. (*B, D*) Relative hepatic mRNA expression of selected genes controlling apoptosis (*B*) and proliferation (*D*) assessed by RT-qPCR. (*C*) Representative liver sections processed for immunohistochemistry/immunofluorescence with anti-BrdU (*brown*) or anti-Ki67 (*red*) antibodies; counterstaining with hematoxylin (anti-BrdU); nuclei stained with DAPI (*blue*; anti-Ki67). Quantification of the staining. Scale bar: 50 μm. Numbers under each bar graph and in the upper right corner of each image indicate the weeks of treatment with *Stk25*-targeting ASOs or placebo. Data are mean ± SEM from 7 to 8 mice per group. Cntr, control; wks, weeks. ^a^*P* < .05 for *Stk25*-targeting ASOs vs PBS; ^b^*P* < .05 for *Stk25* ASO vs Cntr ASO; ^c^*P* < .05 for GalNAc-*Stk25* ASO vs GalNAc-cntr ASO.

**Figure 17:**
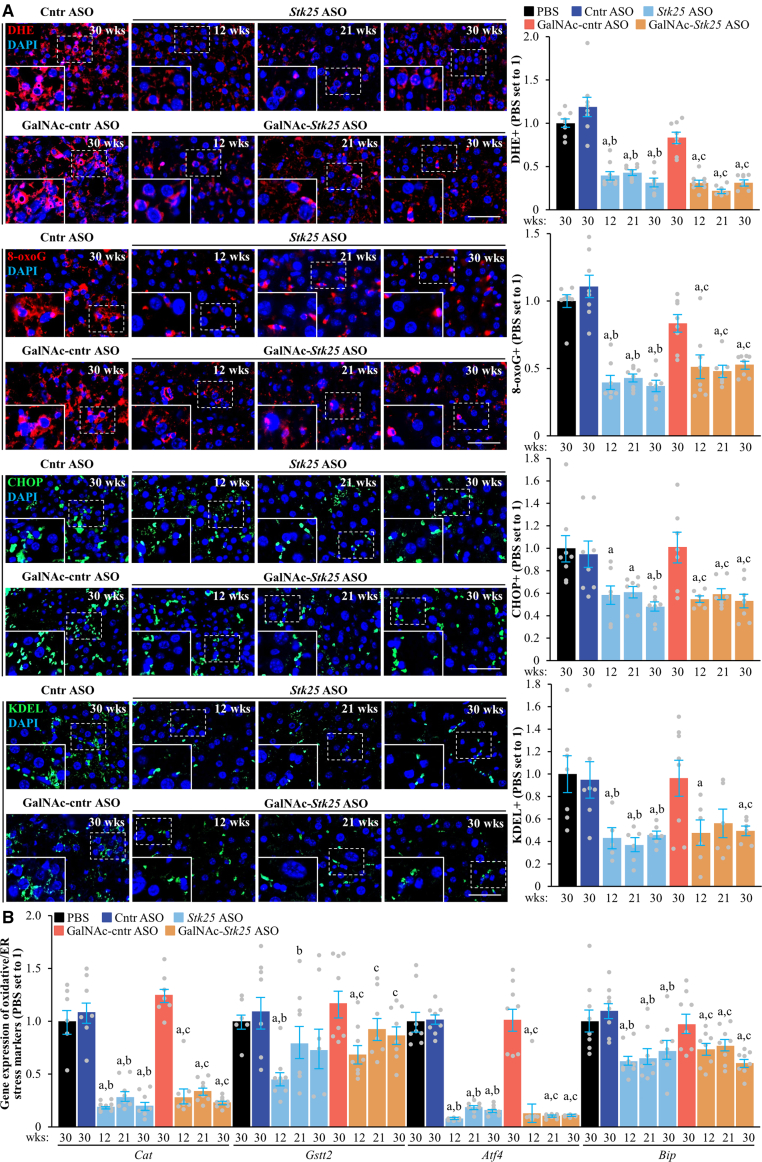
**Administration of *Stk25*-targeting ASOs alleviates steatotoxicity-related oxidative and ER stress in the livers of mice with experimentally induced MASH-driven HCC.** (*A*) Representative liver sections stained with DHE (*red*) or processed for immunofluorescence with anti-8-oxoG (*red*), anti-CHOP (*green*), or anti-KDEL (*green*) antibodies; nuclei stained with DAPI (*blue*). Quantification of the staining. Scale bar: 50 μm. (*B*) Relative hepatic mRNA expression of selected genes controlling oxidative and ER stress assessed by RT-qPCR. Numbers under each bar graph and in the upper right corner of each image indicate the weeks of treatment with *Stk25*-targeting ASOs or placebo. Data are mean ± SEM from 7 to 8 mice per group. Cntr, control; wks, weeks. ^a^*P* < .05 for *Stk25*-targeting ASOs vs PBS; ^b^*P* < .05 for *Stk25* ASO vs Cntr ASO; ^c^*P* < .05 for GalNAc-*Stk25* ASO vs GalNAc-cntr ASO.

Finally, we aimed to identify the possible mechanisms linking MASH features and tumor formation. We found that mice treated with *Stk25*-targeting ASOs had less apoptosis in the livers, as indicated by a lower number of TUNEL-labeled hepatocytes and decreased mRNA expression of several pro-apoptotic mediators (Figure 16*A–B*). This was associated with diminished compensatory proliferation, evidenced by a reduced number of bromodeoxyuridine (BrdU)- and Ki67-positive cells as well as suppressed expression of proliferation markers (Figure 16*C–D*). We also found that mice receiving *Stk25* ASO or GalNAc-*Stk25* ASO displayed lower levels of hepatic oxidative stress measured by dihydroethidium (DHE) staining for superoxide radicals (O_2_^•−^) and immunofluorescence analysis of 8-oxoguanine (8-oxoG) for oxidative DNA damage, as well as less hepatic endoplasmic reticulum (ER) stress assessed by immunolabeling for C/EBP homologous protein (CHOP; also known as DDIT3; an ER stress-induced apoptosis regulator) and KDEL (an ER retrieval motif) (Figure 17*A*). Consistently, the mRNA abundance of several oxidative/ER stress indicators was significantly decreased in the livers of mice dosed with *Stk25*-targeting ASOs compared with mice in the control groups (Figure 17*B*).

### Antagonizing STK25 Signaling Mitigates Tumorigenicity and Impacts Mitochondrial and Lipid Droplet Dynamics of Human Hepatoma Cells

We next compared the cell-autonomous mode of action of STK25 antagonism to that of 2 anti-HCC drugs approved by the United States Food and Drug Administration (FDA)/European Medicines Agency (EMA): small molecule multi-kinase inhibitors sorafenib (the first-line standard of care for advanced HCC [stage C]) and regorafenib (the second-line therapy for sorafenib-resistant HCC).^2^ Well-differentiated (Hep3B) and poorly differentiated (SNU-475) human HCC cells were treated with *STK25* small interfering RNA (siRNA), 5 μmol/L sorafenib, and/or 5 μmol/L regorafenib (the dosages of drugs were selected to be equivalent to the steady-state plasma concentrations of clinically effective doses^32^, ^33^, ^34^).

**Figure 18:**
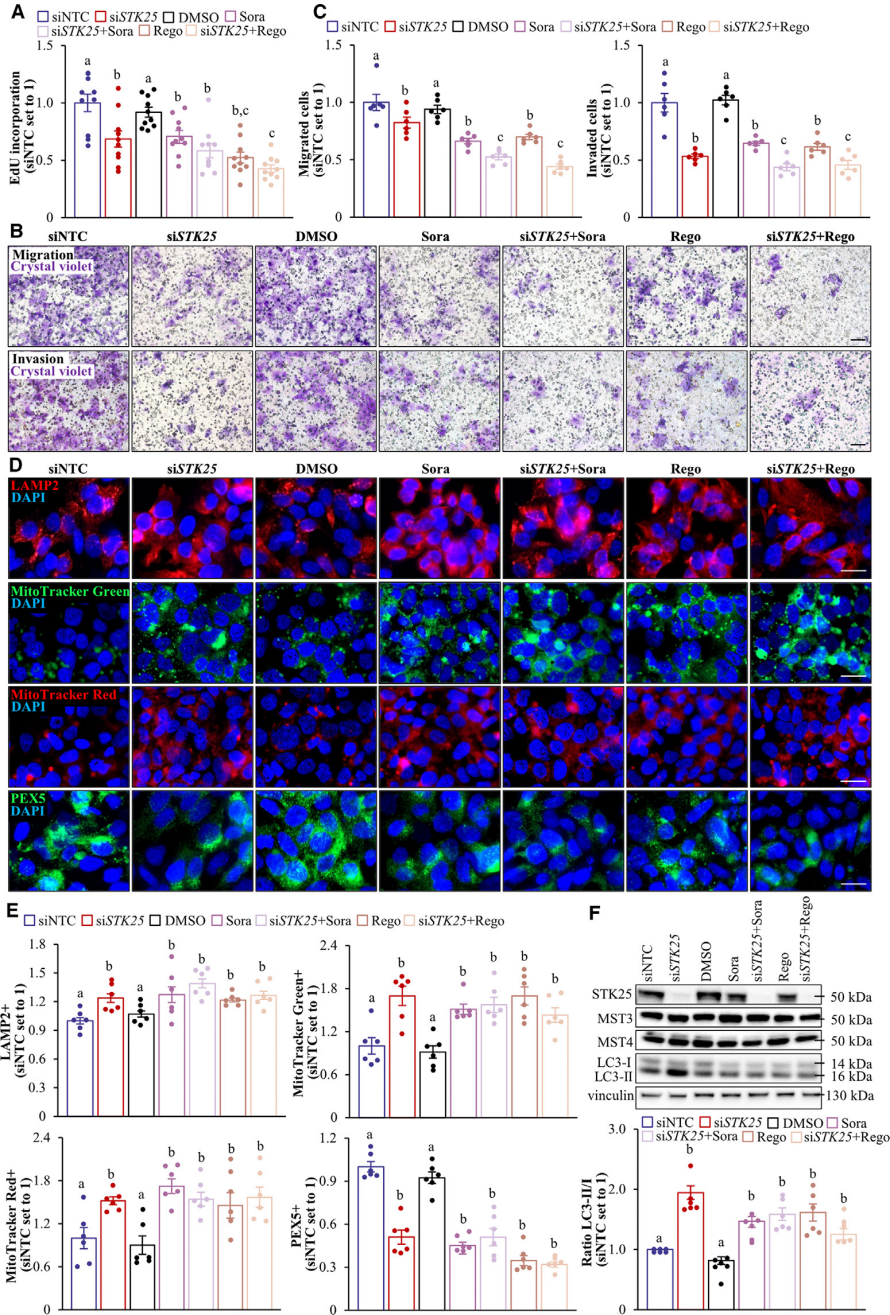
**Antagonizing STK25 signaling mitigates tumorigenicity of human hepatoma cells.** Hep3B cells were incubated with human *STK25* siRNA, 5 μmol/L sorafenib, and/or 5 μmol/L regorafenib. (*A*) Cell proliferation assessed by EdU incorporation. (*B*) Representative images of crystal violet-stained migrated and invaded cells from transwell assay without and with Matrigel pre-coating, respectively. Scale bar: 100 μm. (*C*) Quantification of the staining presented in (*B*). (*D*) Representative images of cells processed for immunofluorescence with anti-LAMP2 (*red*) or anti-PEX5 (*green*) antibodies or stained with MitoTracker Green (*green*) or MitoTracker Red (*red*); nuclei stained with DAPI (*blue*). Scale bar: 25 μm. (*E*) Quantification of the staining presented in (*D*). (*F*) Cell lysates were analyzed by Western blot using antibodies specific for MST3, MST4, LC3, or STK25. Protein levels were assessed by densitometry; representative Western blots are shown with vinculin used as a loading control. Data are mean ± SEM from 6 (*B–F*) or 10 (*A*) wells per group. NTC, non-targeting control; Rego, regorafenib; Sora, sorafenib. Different letters above each bar indicate significant differences at *P* < .05.

**Figure 19:**
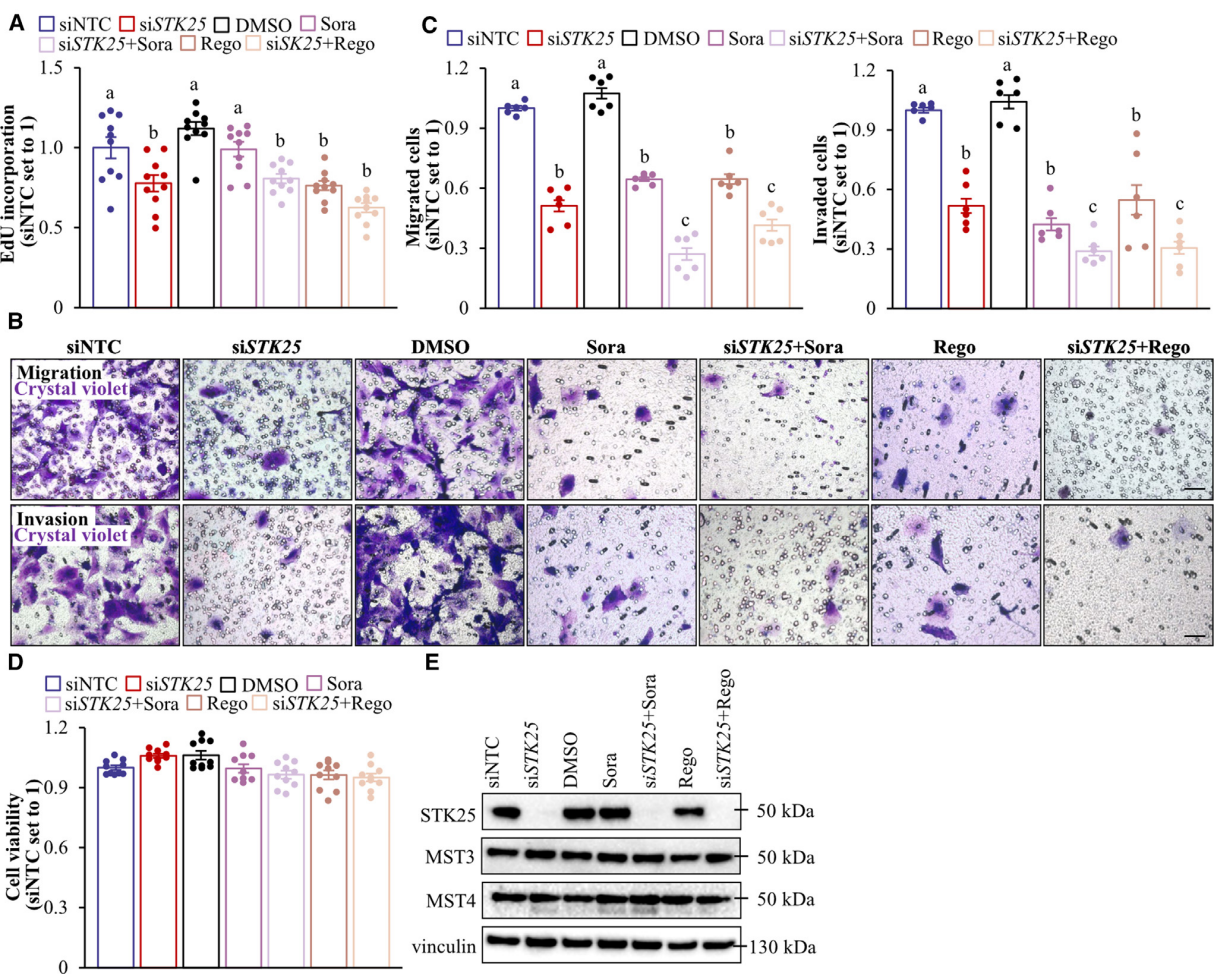
**Analysis of tumorgenicity of SNU-475 cells.** SNU-475 cells were treated with human *STK25* siRNA, 5 μmol/L sorafenib, and/or 5 μmol/L regorafenib. (*A*) Cell proliferation assessed by EdU incorporation. (*B*) Representative images of crystal violet-stained migrated and invaded cells from transwell assay without and with Matrigel pre-coating, respectively. Scale bars: 50 μm. (*C*) Quantification of the staining presented in (*B*). (*D*) Cell viability measured by colorimetric assay. (*E*) Cell lysates were analyzed by Western blot using antibodies specific for STK25, MST3, or MST4. Representative Western blots are shown with vinculin used as a loading control. Data are mean ± SEM from 6 (*B–C*) or 10 (*A, D*) wells per group. NTC, non-targeting control; Rego, regorafenib; Sora, sorafenib. Different letters above the bars indicate significant differences at *P* < .05.

**Figure 20:**
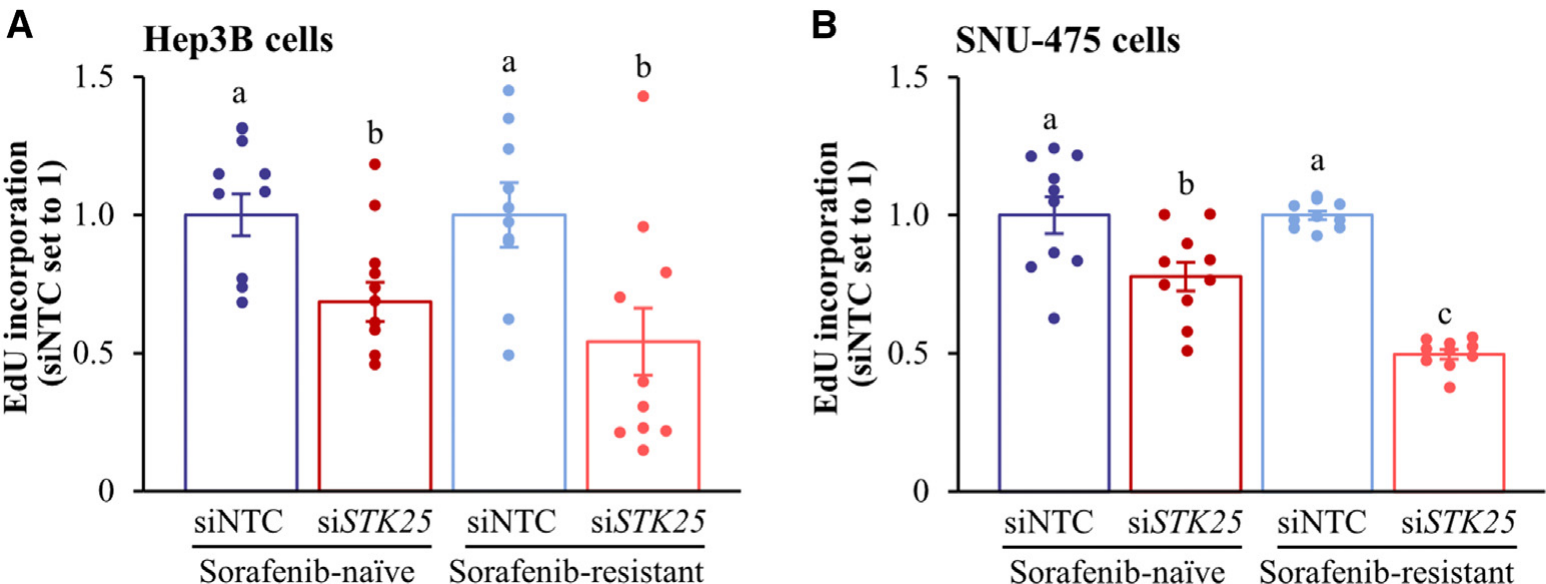
**Assessment of proliferation of sorafenib-resistant hepatoma cells.** Sorafenib-resistant Hep3B cells (*A*) and SNU-475 cells (*B*) were generated by incubation with sorafenib at concentrations of 10 μmol/L and 15 μmol/L, respectively, for 3 rounds of 72 hours each as described.^38^^,^^39^ Cell proliferation in sorafenib-resistant hepatoma cells was assessed by EdU incorporation and data of sorafenib-naïve cells are included for reference. Data are mean ± SEM from 10 wells per group, with all siNTC groups set to 1. NTC, non-targeting control. Different letters above each bar indicate significant differences at *P* < .05.

**Figure 21:**
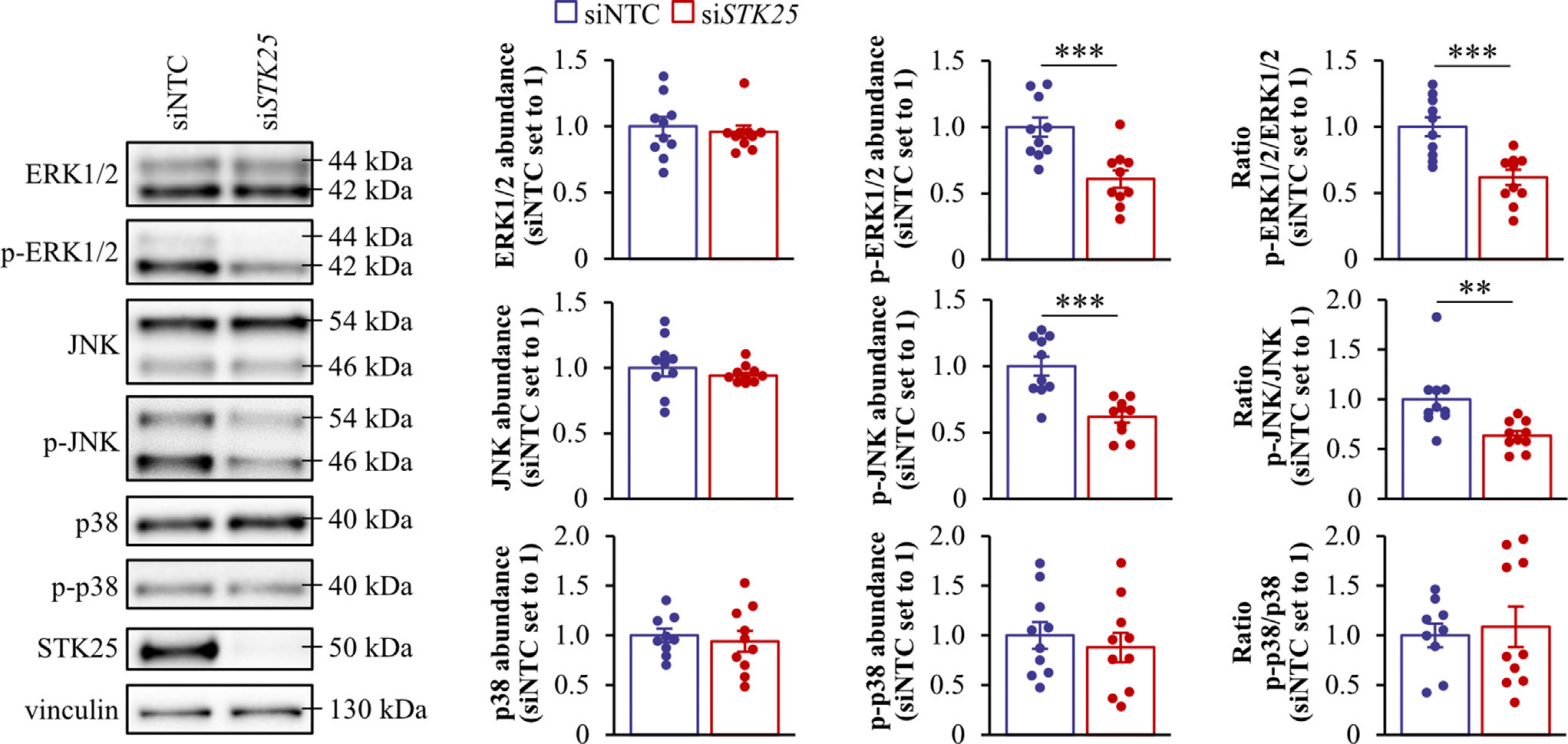
**Analysis of pro-tumorigenic signaling pathways in human hepatoma cells.** Hep3B cells were transfected with human *STK25* siRNA or non-targeting control siRNA. Cell lysates were analyzed by Western blot using antibodies specific for ERK1/2, phospho-ERK1/2 (Thr^202^/Tyr^204^), JNK, phospho-JNK (Thr^183^/Tyr^185^), p38, phospho-p38 (Thr^180^/Tyr^182^), or STK25. Protein levels were assessed by densitometry; representative Western blots are shown with vinculin used as a loading control. Data are mean ± SEM from 9 to 10 wells per group. NTC, non-targeting control. ∗∗*P* < .01; ∗∗∗*P* < .001.

**Figure 22:**
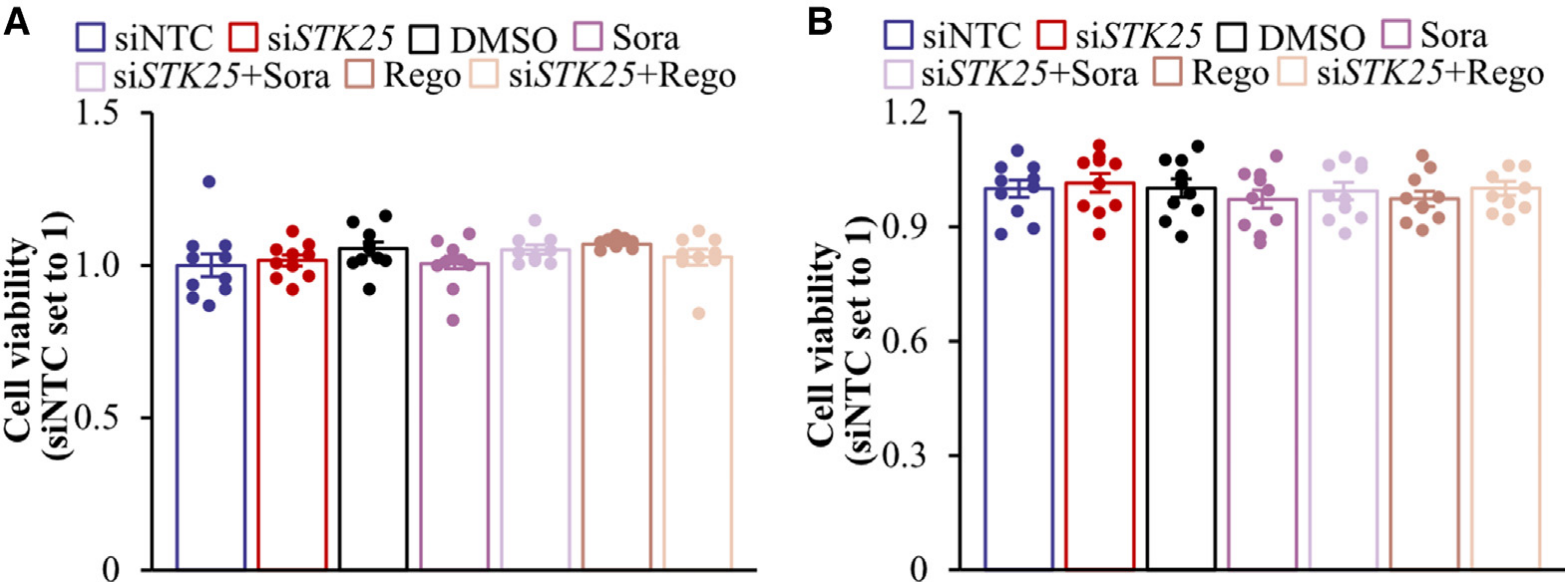
**Measurement of viability of Hep3B cells.** Hep3B cells were incubated with human *STK25* siRNA, 5 μmol/L sorafenib, and/or 5 μmol/L regorafenib, and cultured without (*A*) or with (*B*) 50 μmol/L oleic acid supplementation. Cell viability was measured by colorimetric assay. Data are mean ± SEM from 10 wells per group. NTC, non-targeting control; Rego, regorafenib; Sora, sorafenib.

We found that Hep3B and SNU-475 cell proliferation, as analyzed by EdU incorporation, was similarly suppressed by *STK25* siRNA, sorafenib, or regorafenib (Figures 18*A*, 19*A*). Of note, STK25 knockdown induced a comparable inhibition of proliferation in sorafenib-naïve and sorafenib-resistant hepatoma cells (Figure 20). We also found that the treatment with *STK25* siRNA, sorafenib, or regorafenib was equally efficient in reducing migration and invasion capacity of Hep3B and SNU-475 cells assessed using transwell assay (Figures 18*B–C*, 19*B–C*). Furthermore, the effect on proliferation, migration, and invasion was enhanced in cells incubated with the combination of *STK25* siRNA and sorafenib or *STK25* siRNA and regorafenib (Figures 18*A–C*, 19*A–C*). Interestingly, the silencing of STK25 in Hep3B cells decreased the activation of mitogen-activated protein kinases (MAPKs), extracellular signal-regulated kinase 1/2 (ERK1/2), and Jun N-terminal kinase (JNK), which are key signaling components promoting proliferation and migration in human HCC,^35^, ^36^, ^37^ whereas the phosphorylation status of p38 MAPK remained unaffected (Figure 21). We detected no difference in viability comparing hepatoma cells that received active treatments vs placebo (Figures 19*D*, 22*A*).

**Figure 23:**
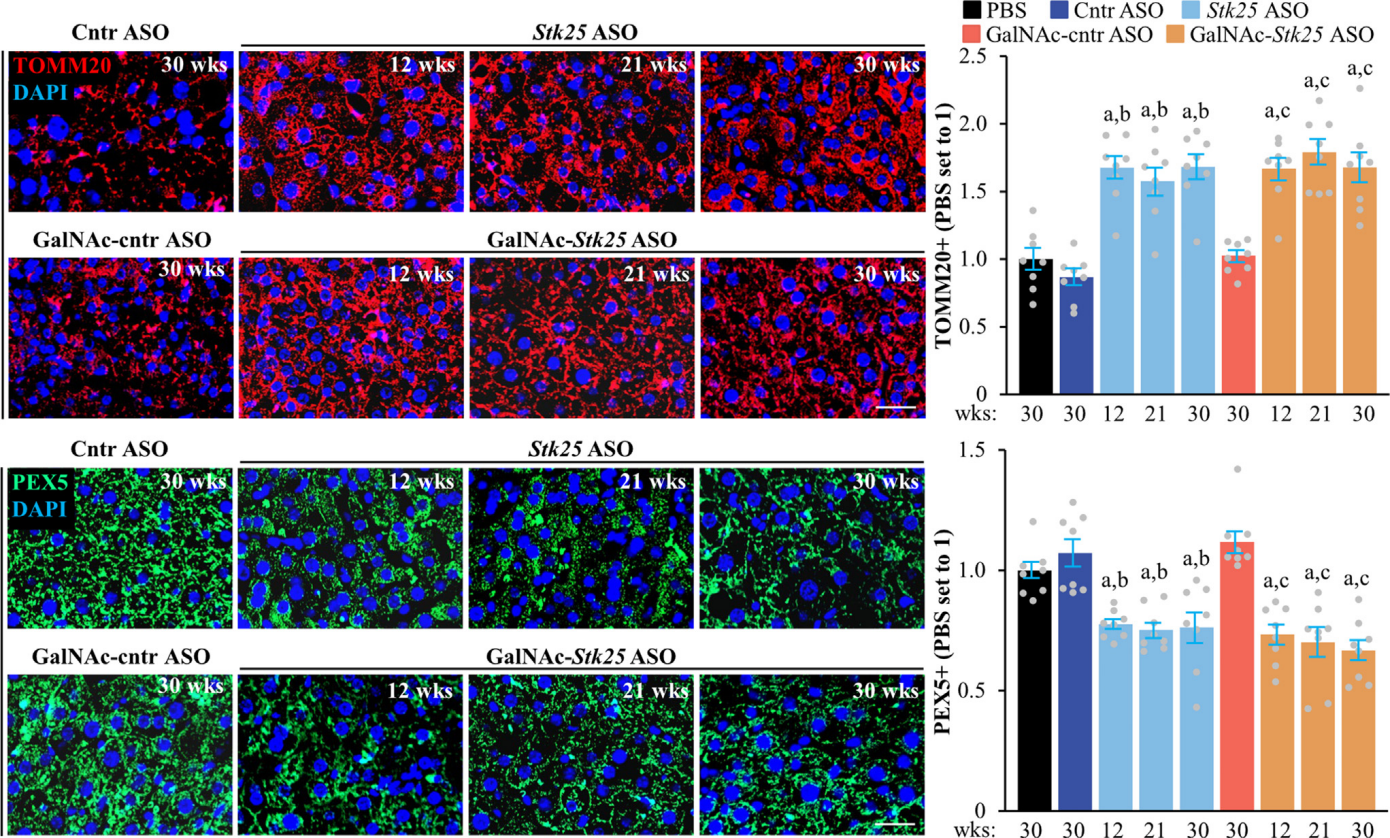
**Analysis of hepatic mitochondrial content and peroxisomal biogenesis in mice with experimentally induced MASH-driven HCC.** Representative liver sections processed for immunofluorescence with anti-TOMM20 (*red*) or anti-PEX5 (*green*) antibodies; nuclei stained with DAPI (*blue*). Quantification of the staining. Scale bar: 50 μm. Numbers under each bar graph and in the upper right corner of each image indicate the weeks of treatment with *Stk25*-targeting ASOs or placebo. Data are mean ± SEM from 8 mice per group. Cntr, control; wks, weeks. ^a^*P* < .05 for *Stk25*-targeting ASOs vs PBS; ^b^*P* < .05 for *Stk25* ASO vs Cntr ASO; ^c^*P* < .05 for GalNAc-*Stk25* ASO vs GalNAc-cntr ASO.

The mode of action of sorafenib and regorafenib involves enhanced hepatocyte autophagic flux.^40^^,^^41^ As expected, Hep3B cells treated with sorafenib or regorafenib displayed higher levels of canonical and alternative autophagy examined by the conversion of LC3I to LC3II and immunofluorescence analysis of lysosome-associated membrane-protein-2 (LAMP2), respectively (Figure 18*D–F*). Intriguingly, the silencing of STK25 induced a comparable upregulation of canonical and alternative autophagy (Figure 18*D–F*). Considering that STK25 regulates hepatocellular lipid homeostasis, which is critical for tumor energy metabolism and biomass synthesis, we next studied mitochondrial and peroxisomal function in Hep3B cells. We discovered that the incubation with *STK25* siRNA, sorafenib, and/or regorafenib led to an equal increase in mitochondrial content and activity quantified by MitoTracker Green (covalently binding mitochondrial matrix proteins) and MitoTracker Red (monitoring mitochondrial membrane potential) staining, respectively (Figure 18*D–E*), which was accompanied by a decrease in peroxisomal biogenesis determined by immunolabeling for PEX5 (Figure 18*D–E*). Sorafenib and regorafenib have also been reported earlier to stimulate mitochondrial respiration in HCC cells^42^; however, the decline in peroxisomal function, to our knowledge, has not been described before. Consistent with our in vitro findings, we observed elevated mitochondrial content and reduced peroxisomal biogenesis in the livers of mice dosed with *Stk25*-targeting ASOs compared with mice in the control groups (Figure 23).

**Figure 24:**
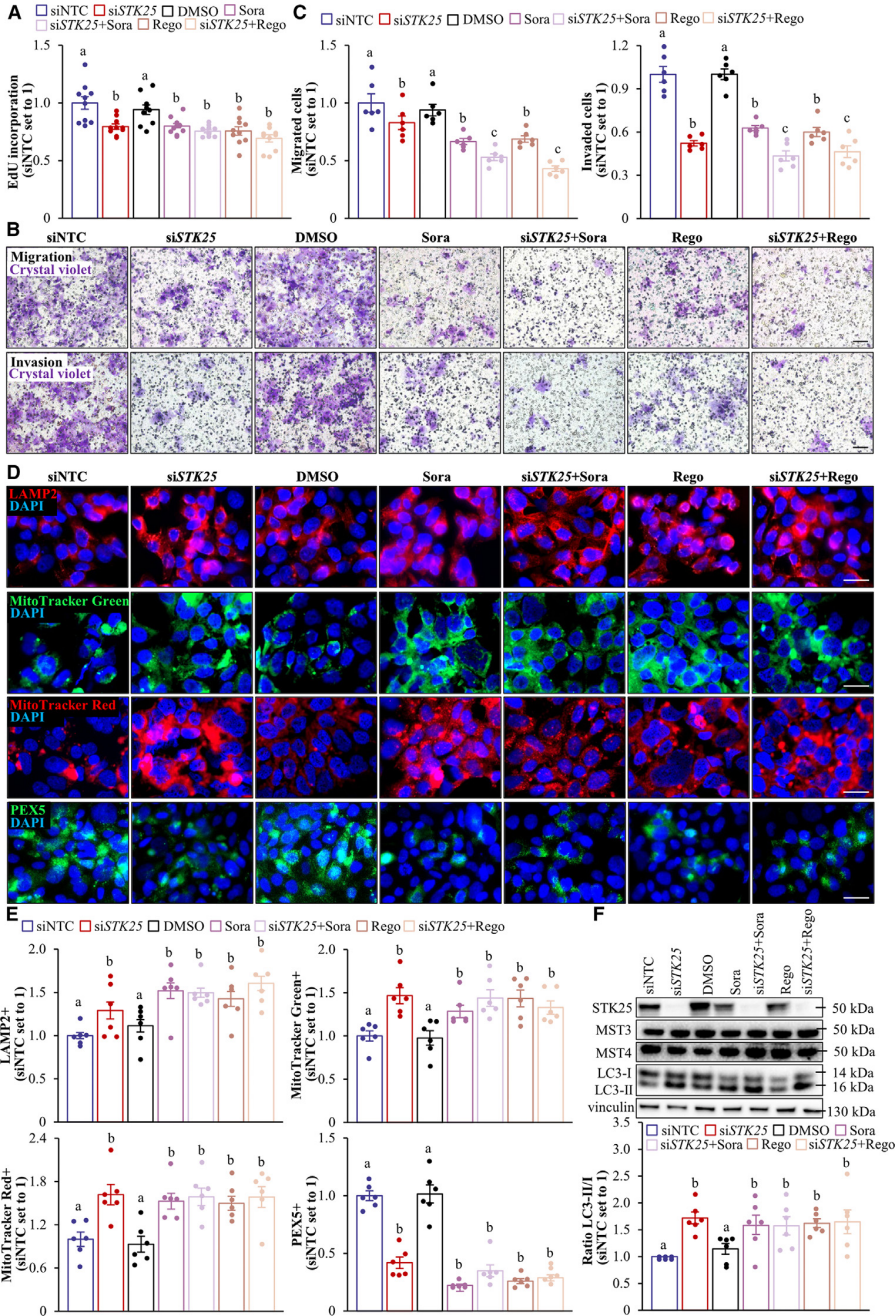
**Assessment of tumorgenicity of oleate-treated Hep3B cells.** Hep3B cells were incubated with human *STK25* siRNA, 5 μmol/L sorafenib, and/or 5 μmol/L regorafenib, and cultured with 50 μmol/L oleic acid supplementation. (*A*) Cell proliferation assessed by EdU incorporation. (*B*) Representative images of crystal violet-stained migrated and invaded cells from transwell assay without and with Matrigel pre-coating, respectively. Scale bar: 100 μm. (*C*) Quantification of the staining presented in (*B*). (*D*) Representative images of cells processed for immunofluorescence with anti-LAMP2 (*red*) or anti-PEX5 (*green*) antibodies or stained with MitoTracker Green (*green*) or MitoTracker Red (*red*); nuclei stained with DAPI (*blue*). Scale bar: 25 μm. (*E*) Quantification of the staining presented in (*D*). (*F*) Cell lysates were analyzed by Western blot using antibodies specific for MST3, MST4, LC3, or STK25. Protein levels were assessed by densitometry; representative Western blots are shown with vinculin used as a loading control. Data are mean ± SEM from 6 (*B–F*) or 10 (*A*) wells per group. NTC, non-targeting control; Rego, regorafenib; Sora, sorafenib. Different letters above each bar indicate significant differences at *P* < .05.

Remarkably, a pattern of changes caused by STK25 knockdown and administration of sorafenib or regorafenib was largely similar in Hep3B cells, which were challenged with oleic acid to replicate the metabolic milieu in high-risk subjects (Figures 22*B*, 24).

**Figure 25:**
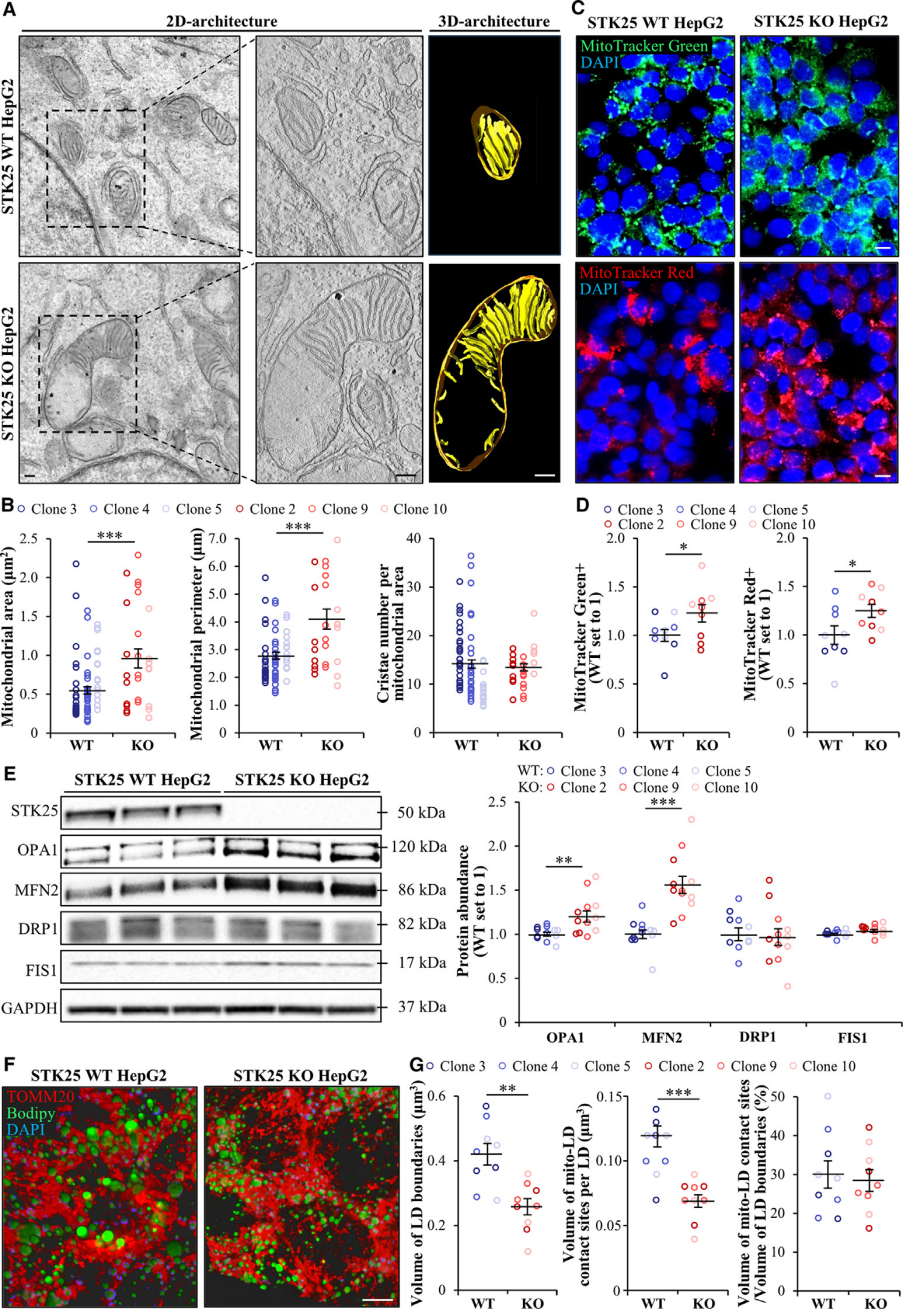
**Loss of STK25 impacts mitochondrial and lipid droplet dynamics of human hepatoma cells.** STK25-depleted and wild-type HepG2 cells were cultured with 50 μmol/L oleic acid supplementation. (*A*) Representative 2D and reconstructed 3D images showing mitochondrial morphology. Scale bar: 200 nm. (*B*) Quantification of mitochondrial size and cristae density based on 2D images presented in (*A*). (*C*) Representative images of cells stained with MitoTracker Green (*green*) or MitoTracker Red (*red*); nuclei stained with DAPI (*blue*). Scale bar: 10 μm. (*D*) Quantification of the staining presented in (*C*). (*E*) Cell lysates were analyzed by Western blot using antibodies specific for OPA1, MFN2, DRP1, FIS1, or STK25. Protein levels were assessed by densitometry; representative Western blots are shown with GAPDH used as a loading control. (*F*) Representative 3D z-stack images of cells stained with Bodipy 493/503 (*green*) and processed for immunofluorescence with anti-TOMM20 (*red*) antibodies; nuclei stained with DAPI (*blue*). Scale bar: 10 μm. (*G*) Quantification of volume of lipid droplets and mitochondria–lipid droplet contact sites based on 3D z-stack images presented in (*F*). Data are mean ± SEM from 3 clones per group. LD, lipid droplet; mito, mitochondria; KO, knockout; WT, wild-type. ∗*P* < .05; ∗∗*P* < .01; ∗∗∗*P* < .001.

To identify the mechanisms of enhanced hepatocyte mitochondrial activity (Figure 18*D–E*), we analyzed mitochondrial ultrastructure by transmission electron microscopy (TEM) in HepG2 where STK25 was depleted by CRISPR/Cas9 editing vs wild-type cells. We detected a significant increase in mitochondrial area and perimeter, without any change in the number of cristae per mitochondrial area, when comparing the hepatoma cells where STK25 was knocked out vs control cells (Figure 25*A–B*; Supplementary Movies 1–2). Notably, most mitochondria in STK25-depleted hepatoma cells exhibited a nonuniform arrangement of cristae, characterized by some regions showing elevated cristae density interspersed with segments devoid of cristae (Figure 25*A*; Supplementary Movies 1–2). In line with our observations in STK25-deficient Hep3B cells (Figure 18*D–E*), we found higher mitochondrial content and activity as evidenced by upregulated staining of MitoTracker Green and Red, respectively, in HepG2 cells where STK25 was knocked out (Figure 25*C–D*). Western blot analysis also demonstrated that the loss of STK25 enhanced the protein abundance of mitochondrial fusion markers optic atrophy 1 (OPA1) and mitofusin 2 (MFN2), without modifying the levels of mitochondrial fission indicators dynamic-related protein (DRP1) and fission 1 (FIS1) (Figure 25*E*).

Next, we tested the hypothesis that antagonizing STK25 signaling augmented mitochondrial activity by increasing the contact between the mitochondrial reticular network and lipid droplets. To this end, we quantified the volume of spatial mitochondria–lipid droplet overlapping zones on 3D images acquired using confocal microscopy of HepG2 cells where neutral lipids were labeled by Bodipy 493/503 and mitochondrial mass by TOMM20. Consistent with our earlier observations,^12^^,^^13^^,^^15^ we detected a decreased volume of lipid droplets in hepatoma cells where STK25 was knocked out (Figure 25*F–G*). Interestingly, the average volume of mitochondria–lipid droplet contact sites per lipid droplet was lower in STK25-depleted hepatoma cells; however, since the size of lipid droplets was significantly reduced, the contact sites as a proportion of lipid droplet volume remained similar in STK25 knockout vs wild-type cells (Figure 25*F–G*).

## Discussion

MASH-associated HCC remains a significant clinical challenge due to the aggressive nature of the disease and its poorly understood molecular basis. This study provides several lines of compelling evidence that antagonizing the signaling of STE20-type kinase STK25 mitigates the initiation and progression of metabolically induced HCC. First, the protein levels of STK25, as well as the abundance of its active form phospho-STK25 (Thr^174^), were increased in human MASH-driven HCC compared with healthy liver tissue. Second, STK25 knockout in human hepatoma cells blocked tumor formation and growth in a xenograft mouse model. Third, *Stk25*-targeting ASOs efficiently suppressed the development and exacerbation of hepatocarcinogenesis in a mouse model of MASH-related HCC. Finally, the in vitro silencing of STK25 alleviated tumorigenicity of human hepatoma cells by inhibiting proliferation, migration, and invasion.

Our previous investigations have shown that the whole-body genetic ablation of STK25 protects mice from hepatocarcinogenesis in the context of MASH.^18^ However, STK25 is ubiquitously expressed, and using a model of global *Stk25*^−/−^ mice presents an intrinsic limitation of STK25 being depleted throughout the development and in all body locations. Here, we assessed the in vivo effect of 2 classes of *Stk25*-targeting ASOs: GalNAc-conjugated *Stk25* ASO (selectively delivered to hepatocytes via the GalNAc-binding asialoglycoprotein receptor [ASGRP]) and the parent unconjugated *Stk25* ASO (broadly distributed to peripheral organs without penetrating the blood-brain barrier). Both ASO types were administered in a mouse model, where MASH-related HCC was triggered by combining an injection with procarcinogen DEN and a high-fat diet feeding. We found that the treatment with GalNAc-*Stk25* ASO achieved similar effectiveness in reducing HCC tumor formation and growth compared with 4-fold higher doses of *Stk25* ASO. This result is aligned with our present and previous studies reporting comparable efficacy of the selected dosing regimen of GalNAc-*Stk25* ASO and *Stk25* ASO in suppressing hepatic STK25 expression by about 90%.^14^^,^^17^ Together, these data suggest that the selective inhibition of STK25 in hepatocytes is sufficient to mitigate metabolically triggered HCC in mice. Notably, the activity of GalNAc-conjugated ASOs is superior to that of the parent unconjugated ASOs in ASGPR^+^ human HCC cells, but not in ASGPR^−^ cells.^43^ Therefore, prescreening patients based on functional ASGPR expression in tumors may be advantageous for GalNAc-*Stk25* ASO to be clinically efficacious.

Patients with MASH-driven HCC tend to be diagnosed at a more advanced stage of tumor progression compared with subjects with other HCC etiologies such as viral hepatitis and excessive alcohol consumption, rendering curative surgery impossible.^8^^,^^44^, ^45^, ^46^ Thus, interventions that provide therapeutic benefits during the late phase of disease are urgently needed to improve patient prognosis and quality of life. Here, we discovered that the delivery of *Stk25*-targeting ASOs, which commenced at the advanced stage of MASH when first HCC tumors had already developed in most mice, resulted in similar efficacy to ASO administration initiated at the early phase of MASLD. This finding provides the first experimental evidence that *Stk25*-targeting ASOs could serve not only for the prevention but also for the treatment of HCC.

In agreement with our earlier studies, we found that the in vitro silencing of STK25 in human hepatoma cells suppressed proliferation, migration, and invasion,^18^ and the effect was equal to that achieved by incubating cells with sorafenib or regorafenib, which are widely used anti-HCC drugs in current clinical practice. Notably, anti-tumorigenic activity was significantly enhanced when sorafenib or regorafenib treatment was combined with STK25 knockdown, and the silencing of STK25 had a similar impact in sorafenib-naïve and sorafenib-resistant hepatoma cells. These data suggest that STK25 and sorafenib/regorafenib likely operate in different signaling pathways, which is not unexpected because sorafenib and regorafenib are tyrosine kinase inhibitors,^47^ whereas STK25 belongs to the serine/threonine kinase family.^48^ Consistently, previous research has demonstrated that STK25 is not directly targeted by sorafenib.^49^ Together, our results warrant further investigations to validate the potential benefits of STK25 inhibitors as an adjunctive therapy to sorafenib and regorafenib, as well as in HCC tumors that are resistant to the current first-line standard of care.

Intriguingly, we detected marked changes in mitochondrial ultrastructure in STK25-depleted human hepatoma cells, including about a 1.5-fold increase in mitochondrial area and perimeter as well as a nonuniform arrangement of cristae, which suggest a shifted equilibrium towards fusion and a higher bioenergetic efficiency of mitochondria.^50^^,^^51^ Consistent with this interpretation, we found an elevated protein abundance of mitochondrial fusion markers, higher mitochondrial membrane potential, and an enhanced mitochondrial β-oxidation^13^^,^^17^ in STK25-deficient hepatocytes. These observations are interesting considering recent evidence that functional impairment in mitochondrial energetics not only contributes to the excessive liver lipid storage during the initial stages of MASLD but also accelerates the transition to MASH and HCC.^52^^,^^53^ Specifically, upregulated mitochondrial fusion is implicated in reducing proliferation, migration, and invasion of human HCC cells.^54^, ^55^, ^56^ In parallel with alterations in mitochondrial dynamics, we discovered a suppression of peroxisomal biogenesis in STK25-deficient hepatoma cells. To this end, hepatic mitochondrial dysfunction at the early phase of MASLD is known to force a higher degree of fatty acid oxidation to peroxisomes, resulting in exaggerated oxidative and ER stress, which, in a vicious cycle, promotes liver inflammation, fibrosis, and hepatocellular apoptosis and proliferation, thereby driving the disease progression towards MASH and ultimately, to HCC.^52^^,^^53^^,^^57^ Together, these data suggest that regulating the homeostatic balance between mitochondrial and peroxisomal fatty acid oxidation likely contributed to the mitigation of hepatocarcinogenesis by STK25 inhibitors.

STK25 belongs to the GCKIII subfamily of STE20-type kinases together with MST3 (also known as STK24) and MST4 (also known as STK26 or MASK).^48^ Similarly to STK25, MST3 and MST4 bind to hepatocellular lipid droplets and have been identified as drivers of hepatic steatotoxicity and tumorigenicity based on expression profiling in liver biopsies from subjects with MASH and MASH-related HCC as well as in vitro investigations in cultured human hepatocytes.^15^^,^^58^, ^59^, ^60^ We and other research groups also reported that, similarly to STK25, the inactivation of MST3 by genetic knockout or by ASO treatment protects mice against diet-induced insulin resistance and MASLD.^61^^,^^62^ In contrast, global MST4 deficiency has no impact on systemic insulin sensitivity or liver steatosis in obese mice.^63^ Importantly, in this study, we did not detect any alterations in the levels of MST3 or MST4 in STK25-deficient mouse livers or human hepatoma cells (Figures 6*B*, 18*F*, 19*E*). Together, these data suggest that the presence of MST3 and MST4 could not compensate for the lack of STK25, implying overlapping but non-redundant roles of GCKIII kinases in the regulation of liver lipid homeostasis and hepatocarcinogenesis.

The present study has some limitations. Here, we examined the impact of pharmacologic STK25 antagonism in a mouse model using the ASOs targeting mouse *Stk25*. STK25 is highly conserved in mice and humans (94% and 99% identity on gene and protein level, respectively); however, additional research applying the ASOs that target human *STK25* sequence in mice where the human *STK25* gene has replaced the mouse counterpart, is essential to gain a better prediction of the expected responses in clinical settings. Nonetheless, the combined analyses in liver biopsies from patients with HCC, human hepatoma cell line-derived xenograft model, and cultured human hepatoma cells performed in this study support the benefits of STK25 inhibition in human MASH-driven HCC.

Although this report compellingly highlights the significant impact of suppressing the STK25 abundance on MASH-related HCC, the underlying mode of action of this kinase remains poorly understood. For instance, we found that both total and phosphorylated STK25 (phospho-STK25 [Thr^174^]; active form) levels were elevated in liver biopsies from subjects with HCC vs nontumor controls; however, it is not known whether the pro-tumorigenic activity of STK25 is dependent on its phosphorylation status. Further studies characterizing the severity of MASH-driven HCC in genetically modified mouse models, in which the Thr^174^ residue of endogenous STK25 is replaced with Ala to prevent the phosphorylation, have the potential to illuminate these critical aspects of its function and will be the focus of our future research.

Since 2007, the FDA has approved 12 anti-HCC drugs, including 5 multi-kinase inhibitors and 7 immune checkpoint blockade therapies; however, these approaches show less effect in patients with MASH-associated HCC.^9^^,^^64^, ^65^, ^66^ Notably, to date, only resmetirom, a liver-targeted thyroid hormone receptor (THR)-beta selective agonist, has been licensed by the FDA for the treatment of MASH.^67^ Nevertheless, remestirom is effective only in <30% of patients at 1-year follow-up, and no long-term data are available.^67^ There is thus a significant unmet medical need for novel therapies for the prevention and treatment of HCC in the context of MASH. Together, the results of our investigations, which combine in vivo analyses in mouse models with expression profiling in human liver biopsies and in vitro assessments in cultured human hepatoma cells, demonstrate that antagonizing STK25 signaling mitigates the development and progression of MASH-driven HCC. Future clinical studies are required to test if these results will translate into therapeutic benefits in patients.

## Materials and Methods

### Patient Cohort

*STK25* mRNA expression was measured by RT-qPCR in interoperative liver biopsies collected from 262 Caucasian individuals (men, n = 157; women, n = 105) undergoing surgery at the Department of General, Visceral, and Transplant Surgery at the University Hospital of Tübingen (Tübingen, Germany). Protein analysis by PLA was carried out in a subgroup of the entire study cohort consisting of 133 subjects (men, n = 87; women, n = 46) for whom there was sufficient tissue available. After food withdrawal overnight, liver biopsies were taken from normal, non-diseased tissue determined by the pathologist during surgery, immediately snap frozen in liquid nitrogen, and stored at −80 °C. The participants tested negative for viral hepatitis and had no liver cirrhosis.

All investigations were approved by the Ethics Committee of the University of Tübingen, Germany (368/2012BO2) and were conducted in compliance with the Declaration of Helsinki. All patients provided written informed consent before enrolling in the study.

### Data Collection From Public Databases

Three HCC datasets (GSE25097, GSE14520, and GSE36376) were downloaded from the GEO database. Additionally, we accessed the whole transcriptome sequencing (RNA-seq) data from the HCC Project in TCGA^68^ and GTEx.^69^ GEO, TCGA, and GTEx databases are publicly accessible resources, and written informed consent was obtained from the patients prior to data collection.

### PLA

The STK25/phospho-STK25 (Thr^174^) PLA probes were constructed with prebiotinylated polyclonal antibodies using the TaqMan Protein Assays Oligo Probe Kit (Thermo Fisher Scientific). The PLA was carried out with the TaqMan Protein Assays Core Reagents Kit with Master Mix (Thermo Fisher Scientific) according to the manufacturer’s instructions. In brief, the PLA working probe solution was mixed with the liver lysates and incubated overnight at 4 °C for binding. Subsequently, ligation reaction was performed at 37 °C for 10 minutes, followed by ligase inactivation using the protease solution at 37 °C for 10 minutes and 95 °C for 5 minutes. Quantitative PCR was conducted with the TaqMan Protein Assay Fast Master Mix and Universal PCR Assay (both from Thermo Fisher Scientific) by incubation at 95 °C for 20 seconds to initiate enzyme activation, prior to 40 cycles of amplification (94 °C for 1 second and 60 °C for 20 seconds). The relative protein expression was calculated as ΔCq by subtracting the Cq value for the biological sample from that of the no protein control.

### CRISPR/Cas9 Editing

To generate stable Cas9-expressing cells, HepG2 cells (RRID: CVCL_0027; human hepatoblastoma cells; American Type Culture Collection) were infected with lentiviral particles produced from the lentiCas9-Blast plasmid (RRID: Addgene_52962; a gift from Feng Zhang),^70^ and were subsequently selected with 10 μg/mL blasticidin for 1 week. To obtain STK25 knockout clones, 2 single guide RNAs (sgRNAs) targeting introns 4 and 6, respectively, that lead to a frameshift mutation caused by excision of exons 5 and 6, were designed with the benchling tool (https://www.benchling.com; see Table 3 for the sgRNA sequences). Following in vitro transcription using the HiScribe T7 Quick High Yield RNA Synthesis Kit (NEB), sgRNAs were purified by the RNase Mini Kit (Qiagen) and transfected into Cas9-expressing HepG2 cells with Lipofectamine Messenger Max (Thermo Fisher Scientific) according to the manufacturer’s instructions. Successful deletion of exons 5 and 6 in batch cultures was verified by PCR amplification of genomic DNA, using primers spanning the excised DNA region (see Table 4 for the primer sequences). Single cell-derived homozygous knockout clones obtained through dilution cloning were genotyped as described above and confirmed by Western blot.

**Table 3:** List of sgRNAs Used for CRISPR/Cas9 Editing

Name	Position in genome	Strand	Sequence^a^	PAM	Specificity score	Efficiency score
STK25-E5U	10278	−1	GCGTCATCCCAGGCTCCACG	TGG	41	67
STK25-E6D	10783	1	CCCTTCTAGACAGGACGTGG	AGG	44	61

PAM, protospacer adjacent motif; sgRNA, single guide RNA.

a GeneBank ID: DQ093965.1.

**Table 4:** Sequences of the Primers Used for Genotyping PCR

Gene	Forward primer sequence	Reverse primer sequence
*STK25*	5′-TGGGGGTGGATGTGGCTCTTGT-3′	5′-TAGCGGTCGATGAGCTCCGTGA-3′

PCR, polymerase chain reaction.

### Generation of ASOs

The ASOs used in this study were 16 nucleotides in length and chemically modified with phosphorothioates in the backbone, 3 of the 2′-4′ constrained ethyl residues at each terminus, and a central deoxynucleotide region of 10 residues (3-10-3 gapmer design). The following ASO sequences were used in this study: *Stk25* ASO 5′-GCATAATCCCCTAGGC-3′ and control ASO 5′-GGCCAATACGCCGTCA-3′. For hepatocyte-targeting ASOs, conjugation of the parent *Stk25* ASO and control ASO to GalNAc was performed as described.^71^ For detailed information about the synthesis and safety analysis of *Stk25* ASO and GalNAc-*Stk25* ASO, see Nuñez-Durán et al^14^ and Cansby et al,^17^ respectively. For all in vivo experiments, ASOs were dissolved in PBS (without Ca^2+^ or Mg^2+^; Invitrogen).

### Mice

#### Xenograft model

A total of 1 × 10^7^ cells (STK25-depleted or wild-type HepG2) were suspended in 100 μL of PBS and injected subcutaneously into the upper flanks of 5-week-old male BALB/c nude mice (RRID: IMSR_JAX:000711; Janvier Labs). Viability of cells was checked with Trypan Blue stain 0.4% (Invitrogen) before and after injection. Following inoculation, mice were fed a high-fat diet (60 kcal% fat, D12492; Research Diets). Tumor dimensions were inspected every 3 to 5 days using calipers, and tumor volume was calculated by the formula V = (length × width^2^)/2. Once xenografts reached a size of ∼12 to 14 mm in the maximal diameter (on day 48 post-injection), the mice were killed, and tumors were excised and photographed.

#### MASH-related HCC model

Male C57BL/6J mice (RRID: IMSR_JAX:000664; Charles River) were housed 3 to 5 per cage in a temperature-controlled (21 °C) facility with a 12-hour light/dark cycle and *ad libitum* access to chow and water. To induce HCC in the context of MASH, a single intraperitoneal injection of DEN (25 mg/kg; N0258; Sigma-Aldrich) was performed in 14-day-old mice. Starting from 4 weeks after DEN injection, mice were fed a high-fat diet (60 kcal% fat, D12492, Research Diets) for 30 weeks. At 6 weeks of age, the weight-matched mice were randomly divided into 3 experimental cohorts that were injected intraperitoneally with *Stk25* ASO (50 mg/kg/wk), control ASO (50 mg/kg/wk), GalNAc-*Stk25* ASO (12.5 mg/kg/wk), GalNAc-control ASO (12.5 mg/kg/wk), or PBS twice weekly for the last 12, 21, or 30 weeks of high-fat diet feeding (see Figure 4*A* for a schematic overview of the experimental design). The body weights were recorded weekly, blood was collected for determination of fasting glucose and insulin at several time points during the course of the study, and GTT and ITT were carried out as previously described.^63^ At the age of 36 weeks, mice were killed by cervical dislocation under isoflurane anesthesia after 4 hours of food withdrawal. To investigate the cell proliferation, mice were subjected to a single intraperitoneal injection with BrdU (100 mg/kg; B5002; Sigma-Aldrich) 2 hours before killing. Blood was obtained by cardiac puncture for assessment of ALT and AST levels. Livers were weighed, and each lobe of liver was photographed. The liver tumor parameters were evaluated as previously reported.^18^ Liver tissues were processed for flow cytometry, harvested for histologic and immunohistochemical/immunofluorescence analysis, or snap frozen in liquid nitrogen and stored at –80 °C for examination of protein and gene expression and biochemical assessments as described below.

The mice used in this study received humane care in accordance with the National Institutes of Health (NIH) recommendations outlined in the *Guide for the Care and Use of Laboratory Animals*. All the in vivo experiments were carried out in compliance with the guidelines approved by the local Ethics Committee for Animal Studies at the Administrative Court of Appeals in Gothenburg, Sweden (approval number 5.8.18-20238/2020 for xenograft model and 5.8.18-17285/2018 for MASH-related HCC model).

### Histologic, Immunohistochemical, and Immunofluorescence Analysis

Mouse liver tissues were fixed in 4% (vol/vol) phosphate-buffered formaldehyde (Histolab Products), embedded in paraffin, and sectioned. Paraffin sections were stained with H&E (Histolab Products) for morphologic analysis or with DHE (Life Technologies) to measure superoxide radical formation. The MAS was performed following the Kleiner/Brunt criteria as previously described.^17^ Apoptotic cells were identified by using the HRP-DAB TUNEL Assay Kit (Abcam). For immunohistochemical or immunofluorescence analysis, paraffin sections were incubated with primary antibodies, followed by incubation with biotinylated or fluorescent-dye-conjugated secondary antibodies (see Table 5 for antibody information). Liver tissue samples were also embedded in optimal cutting temperature (OCT) mounting medium (Histolab Products) and frozen in liquid nitrogen, followed by cryosectioning. Cryosections were stained with Bodipy 493/503 (Invitrogen) to assess neutral lipids. Images were acquired using a Zeiss Axio Observer microscope with the ZEN Blue software (RRID: SCR_013672; Zeiss). The total area labeled was quantified in 6 randomly selected microscopic fields (×100 or ×200; distributed over 3 non-consecutive liver sections) per mouse using the ImageJ software (RRID: SCR_003070; NIH).

**Table 5:** List of Antibodies Used for Western Blot, Immunohistochemical/Immunofluorescence Analysis, and Flow Cytometry

Type	Antibody name and catalog number	Working dilution	Company
Primary antibody	anti-STK25 (25821-1-AP)	1:750	Proteintech (Chicago, IL)
	anti-AFP (ab46799)	1:200	Abcam (Cambridge, UK)
	anti-YAP (ab205270)	1:500	Abcam
	anti-GRP78 (sc-166490)	1:250	Santa Cruz Biotechnology (Santa Cruz, CA)
	anti-EpCAM (ab213500) anti-F4/80 (MCA497GA) anti-collagen IV (ab6586)	1:100 1:250 1:200	Abcam Bio-Rad (Hercules, CA) Abcam
	anti-fibronectin (F3648) anti-αSMA (ab5694) anti-ubiquitin (ab7780)	1:300 1:200 1:200	Sigma-Aldrich (St. Louis, MO) Abcam Abcam
	anti-BrdU (ab6326)	1:100	Abcam
	anti-Ki67 (14-5698-82)	1:200	Invitrogen (Waltman, MA)
	anti-8-oxoG (ab62623)	1:500	Abcam
	anti-CHOP (MA1-250) anti-KDEL (ab176333)	1:200 1:500	Invitrogen Abcam
	anti-ERK1/2 (#9102)	1:1000	Cell Signaling Technology (Boston, MA)
	anti-p-ERK1/2 (#9101)	1:1000	Cell Signaling Technology
	anti-JNK (#9252)	1:1000	Cell Signaling Technology
	anti-p-JNK (#4668)	1:500	Cell Signaling Technology
	anti-p38 (#9212)	1:1000	Cell Signaling Technology
	anti-p-p38 (#9211)	1:500	Cell Signaling Technology
	anti-LC3 (#2775) anti-LAMP2 (PA1-655)	1:1000 1:1000	Cell Signaling Technology Invitrogen
	anti-PEX5 (PA5-58716)	1:500	Invitrogen
	anti-TOMM20 (ab186734)	1:50	Abcam
	anti-MST3 (#3723)	1:1000	Cell Signaling Technology
	anti-OPA1 (612607)	1:1000	BD Bioscience (Franklin Lakes, NJ)
	anti-MFN2 (#9482)	1:1000	Cell Signaling Technology
	anti-DRP1 (#8570)	1:1000	Cell Signaling Technology
	anti-FIS1 (#32525)	1:1000	Cell Signaling Technology
	anti-MST4 (#3822) anti-GAPDH (sc-47724) anti-vinculin (sc-25336)	1:1000 1:1000 1:500	Cell Signaling Technology Santa Cruz Biotechnology Santa Cruz Biotechnology
Secondary antibody	Alexa Fluor-488-labeled anti-mouse IgG (A21202)	1:500	Invitrogen
	Alexa Fluor-488-labeled anti-rabbit IgG (A11008)	1:500	Invitrogen
	Alexa Fluor-555-labeled anti-rabbit IgG (A21428)	1:500	Invitrogen
	Alexa Fluor-594-labeled anti-rabbit IgG (A21207) Alexa Fluor-594-labeled anti-rat IgG (A11007)	1:500 1:500	Invitrogen Invitrogen
	Biotinylated-labeled anti-rabbit IgG (E0432)	1:300	Dako (Carpenteria, CA)
	Biotinylated-labeled anti-mouse IgG (E0464)	1:300	Dako
	Biotinylated-labeled anti-rat IgG (BA4001)	1:300	Vector (Burlingame, CA)
	anti-rabbit IgG (#7074)	1:1000	Cell Signaling Technology
	anti-mouse IgG (#7076)	1:1000	Cell Signaling Technology
Fluorophore-conjugated antibody	BV711 anti-CD45 (563709) BV510 anti-F4/80 (123135) BV786 anti-CD11b (740861)	1:400 1:200 1:400	BD Bioscience Biolegend (San Diego, CA) BD Bioscience

### Biochemical Assays

The fasting blood glucose and plasma insulin levels in mice were analyzed by Accu-Chek glucometer (Roche Diagnostics) and the Ultra-Sensitive Mouse Insulin enzyme-linked immunosorbent assay (ELISA) Kit (Crystal Chem), respectively. Hepatic TAG content was measured using the Triglyceride Colorimetric Assay Kit (Cayman Chemical). Plasma ALT and AST concentrations were assessed with the Mouse Alanine Aminotransferase ELISA Kit and the Mouse Aspartate Aminotransferase ELISA Kit (both from MyBioSource), respectively. All biochemical assays were performed in duplicate.

### Flow Cytometry

Fresh liver tissues from mice were digested with 1 mg/mL collagenase type IV (Abcam) at 37 °C for 30 minutes. The supernatants were passed through a 100-μm mesh filter (Thermo Fisher Scientific), followed by density gradient centrifugation using Lymphocyte Separation Medium (MP Biomedicals) to collect liver-infiltrating lymphocytes. The isolated cells were incubated with blocking buffer (5 mmol/EDTA, 1% bovine serum albumin [BSA], and 0.05% NaN_3_ in PBS) and then labeled with fluorophore-conjugated antibodies at 4 °C for 1 hour in darkness (see Table 5 for antibody information). Stained cells were acquired on the BD LSRFORTESSA X-20 instrument (BD Biosciences) and were analyzed with the FlowJo software (RRID: SCR_008520; Tree Star).

### Cell Culture, Transient Transfections, and Incubation with Anti-HCC Drugs

Hep3B and SNU-475 (RRID: CVCL_0326 and RRID: CVCL_0497, respectively; human HCC cells; American Type Culture Collection) were cultured as previously described.^60^ For RNA interference, Hep3B/SNU-475 cells were transfected with human *STK25* siRNA (s20570; Ambion) or scrambled siRNA (SIC001; Sigma-Aldrich) using Lipofectamine RNAiMax (Thermo Fisher Scientific). Twenty-four hours after transfections, the culture medium was replaced by fresh medium, with or without supplementation of 50 μmol/L oleic acid (Sigma-Aldrich), for a subsequent 48-hour incubation. The cells were also treated with 5 μmol/L sorafenib (Cayman Chemical), 5 μmom/L regorafenib (Selleck Chemicals), or vehicle control (dimethylsulfoxide [DMSO]; Invitrogen) for an additional 24 hours prior to harvest. HepG2 (STK25-depleted and wild-type clones, see the “CRISPR/Cas9 Editing” section above) were maintained in Dulbecco's Modified Eagle’s Medium (DMEM; GlutaMAX supplemented; Gibco) supplemented with 10% (vol/vol) fetal bovine serum (FBS) and 1% (vol/vol) penicillin/streptomycin (Gibco), and were challenged with 50 μmol/L oleic acid for 48 hours before further processing.

### Evaluation of Tumorigenicity of Human Hepatoma Cells

Cell viability was analyzed using the CellTiter-Blue Cell Viability Assay (Promega) according to the manufacturer’s protocol. The proliferation was measured by the Click-iT EdU Proliferation Assay for Microplates Kit (Thermo Fisher Scientific). Transwell assay was performed to determine migration/invasion as previously described.^60^

Cells were stained with MitoTracker Green and Red (both from Thermo Fisher Scientific) as described earlier.^72^ In parallel, cells were processed for immunofluorescence with anti-LAMP2 or anti-PEX5 primary antibodies, followed by incubation with fluorescent-dye-conjugated secondary antibodies (see Table 5 for antibody information). Images were acquired using a Zeiss Axio Observer microscope with the ZEN Blue software (Zeiss). The total area labeled was quantified in 6 randomly selected microscopic fields (×100 or ×200) per well of cell culture chamber using the ImageJ software.

### Transmission Electron and Confocal Microscopy

To analyze mitochondrial ultrastructure, cells were immersed in Karnovsky fixative at room temperature for 1 hour, post-fixed at room temperature for 1 hour with 1% osmium tetra-oxide and 1% potassium ferrocyanide in 0.05 mol/L cacodilate buffer, washed, and then stained with 1% uranyl acetate at room temperature for 30 minutes. Following dehydration through a series of increasing ethanol concentrations, cells were embedded in epoxy Hard-Plus resin (EMS) and polymerized at 60 °C for 16 hours. Ultrathin sections were obtained using an ultra-microtome (UC6, Leica Microsystem). Mesh grids with 70-nm sections were imaged using a TEM Talos L120C operating at 120 eKv equipped with a BM-Ceta CMOS 4K×4K CCD camera (ThermoFisher). Images were captured in focus at various magnifications, and mitochondrial size was analyzed using the ImageJ software. Three-dimensional tomography was performed as previously reported.^73^ Briefly, tilt series (±65° with 1° increments) of the grids were collected on the TEM Talos L120C at a nominal magnification of ×22,000 with a pixel size of 0.634 nm/pixel. The tomographic tilt series were aligned and reconstructed by eTomo (University of Colorado). A 3D volume model was drawn following the outline of the outer membranes and cristae of mitochondria using the IMOD package (RRID: SCR_003297; http://bio3d.colorado.edu/imod/).^74^^,^^75^

To investigate the contact between mitochondrial reticular network and lipid droplets, cells were stained with Bodipy 493/503 and processed for immunofluorescence with anti-TOMM20 antibodies (see Table 5 for antibody information) to visualize neutral lipids and mitochondria, respectively. Image stacks were acquired on a Leica SP8 confocal microscope with a ×63/1.4 numerical aperture oil immersion objective (Leica), followed by deconvolution using the Huygens Professional package (RRID: SCR_014237; Scientific Volume Imaging) and analysis with the ImageJ software.^76^

### Western Blot and RT-qPCR

Western blot analysis was carried out as earlier described^77^ (see Table 5 for antibody information). RNA was isolated from human liver biopsies and mouse liver tissues by the RNeasy Tissue Kit (Qiagen) and the EZNA Total RNA Kit (Omega Bio-Tek), respectively. cDNA was synthesized using the High-Capacity cDNA Reverse Transcription Kit (Thermo Fisher Scientific). Relative quantification was performed with the LightCycler480 (Roche Diagnostics) or the CFX Connect Real-Time System (Bio-Rad). The relative quantities of the target transcripts were calculated from duplicate samples after normalization of the data to the endogenous control, *RSP13* (used for human liver biopsies; TIB Molbiol Syntheselabor GmbH) or *β-actin* (used for mouse liver tissues; Thermo Fisher Scientific). The sequences of custom-designed primers used for the analysis of human liver biopsies are listed in Table 6.

**Table 6:** Sequences of the Custom-designed Primers Used for RT-qPCR

Gene	Forward primer sequence	Reverse primer sequence
*COL1A1*	5′-CTGGACCTAAAGGTGCTGCT-3′	5′-GCTCCAGCCTCTCCATCTT-3′
*RPS13*	5′-CCCCACTTGGTTGAAGTTGA-3′	5′-ACACCATGTGAATCTCTCAGGA-3′
*STK25*	5′-GCTCAGCACTGGACTTGCTT-3′	5′-GCAGGATCGTGGCAATGTAT-3′
*TGFB1*	5′-ACTACTACGCCAAGGAGGTCAC-3′	5′-TGCTTGAACTTGTCATAGATTTCG-3′

RT-qPCR, reverse transcription quantitative polymerase chain reaction.

### Statistical Analysis

Unpaired 2-tailed Student’s *t*-test or 1-way analysis of variance (ANOVA) followed by Tukey’s post hoc test were applied when comparing 2 or more groups, respectively. Differences were considered statistically significant at *P* < .05. The Spearman correlation analysis was performed to determine the relationship between the protein abundance of STK25 and phospho-STK25. All statistical analyses were carried out using SPSS statistics (RRID: SCR_002865; IBM Corporation).

The HCC samples extracted from TCGA were divided into high- and low-expressing groups based on the median expression value of *STK25* in all samples. The survival data of patients with HCC from the TCGA was evaluated using the “survival” R package (RRID: SCR_021137; statistical analysis of survival data) and “survminer” R package (RRID: SCR_021094; visualization)^78^ for the prognostic analysis according to the Kaplan-Meier method.
